# Composition and natural history of a snake community from the southern Cerrado, southeastern Brazil

**DOI:** 10.3897/zookeys.1056.63733

**Published:** 2021-08-19

**Authors:** Bruno F. Fiorillo, Jorge Henry Maciel, Marcio Martins

**Affiliations:** 1 Programa de pós-graduação em Ecologia Aplicada, Escola Superior de Agricultura Luiz de Queiroz, Universidade de São Paulo, 13418-900, Piracicaba, SP, Brazil Universidade de São Paulo Piracicaba Brazil; 2 Herp Trips, RPPN Trápaga, Rodovia SP-139, km 80, Zona Rural, S/N – Abaitinga, São Miguel Arcanjo, São Paulo, Brazil Herp Trips São Paulo Brazil; 3 Departamento de Ecologia, Instituto de Biociências, Universidade de São Paulo, 05508-090, São Paulo, SP, Brazil Universidade de São Paulo São Paulo Brazil

**Keywords:** Behaviour, diet, habitat, reproduction, savanna, Serpentes

## Abstract

The natural history of a cerrado snake community in a protected area in southeastern Brazil (Santa Bárbara Ecological Station; SBES) is described. A visual guide and an identification key are also provided to assist researchers and local people in identifying snakes in that region.

Sampling was performed through pitfall traps, time-constrained search, accidental encounters, and observations by local people for two years, which corresponded to 240 days of sampling. Among the 388 individuals found in the field, 33 snake species belonging to 21 genera of seven families were recorded. Most species were restricted or found at least once in non-forest vegetation types (campo sujo, campo cerrado, and cerrado sensu stricto) and a few were restricted to forest habitats (cerradão). Our results show that most species (1) occupy open areas; (2) present both diurnal and nocturnal activity; (3) are primarily terrestrial; (4) include lizards, mammals and/or anurans in the diet; (5) present seasonal reproductive activity; and (6) use mainly visually oriented defensive tactics. Despite its small size (3,154 ha), the SBES harbours preserved habitats and a rich and typical Cerrado snake fauna, including threatened species. Furthermore, most of the SBES snakes occur in non-forest environments (54%) and some species are sensitive to habitat disturbance.

## Introduction

The gathering of information related to natural history, what the organisms do in their respective environments, including the interactions between them (Greene 1994), contributes to the understanding of the functioning of ecosystems and, consequently, to many aspects of conservation, management, and even the appreciation of nature (Caughley 1994; Brooks and McLennan 2002; Dayton 2003). However, even though its relevance is obvious, there is still a large gap in knowledge about the ecology and behaviour of most extant taxa, even in the best-studied regions of the planet (Greene 2005). This type of information is available for only a small fraction of species, usually large or common, and relatively easily studied (Greene 1994).

Despite the high diversity of neotropical snakes (Cadle and Greene 1993; Martins and Oliveira 1998; Sawaya et al. 2008; Guedes et al. 2018; Nogueira et al. 2019), even the most basic information about their natural history is still scarce for most species (Sazima and Haddad 1992; Sawaya et al. 2008; Guedes et al. 2014). In addition, although the number of studies on snake communities from non-forest vegetation types like those of the Caatinga, Cerrado, and Pantanal has increased considerably in recent decades (e.g., Strüssmann and Sazima 1993; Sawaya et al. 2008; Rocha and Prudente 2010; Mesquita et al. 2013a; Guedes et al. 2014), much primary information about the herpetofauna of these areas is still lacking (Colli et al. 2002). In southeastern Brazil, the Cerrado have suffered an extensive loss during the 20^th^ century, mainly due to agricultural and livestock practices. Currently, < 0.8% of the Cerrado original vegetation remains in the state of São Paulo (Kronka et al. 2005); therefore, studies should urgently be carried out in these remnant areas.

Several protected areas in the southern portion of the Cerrado include a mosaic of typical vegetation types of the biome (from grasslands to woodlands), which has contributed to maintaining a high species diversity of amphibians and reptiles within their limits (Sawaya et al. 2008; Araujo et al. 2010, 2013, 2014; Araujo and Almeida-Santos 2011). Thus, these protected areas may guarantee the persistence of populations of several species, contributing to the conservation of this rich fauna.

Herein we provide basic natural history information for a Cerrado snake community inhabiting a protected area, Santa Bárbara Ecological Station, in southeastern Brazil. The snake fauna of this area was previously studied by Araujo et al. (2010), who listed 21 species, 18 of which were found in the field and three from museum records for the Municipality of Águas de Santa Bárbara, where the reserve is located. Here we provide information for 33 species of snakes found during our study. For each species, we provide primary information on habitat and micro-habitat use, time of activity, feeding habits, reproduction, and defence. We also provide a short review of the natural history of each species based on our observations and on previously published accounts.

## Materials and methods

**Figure 1.**
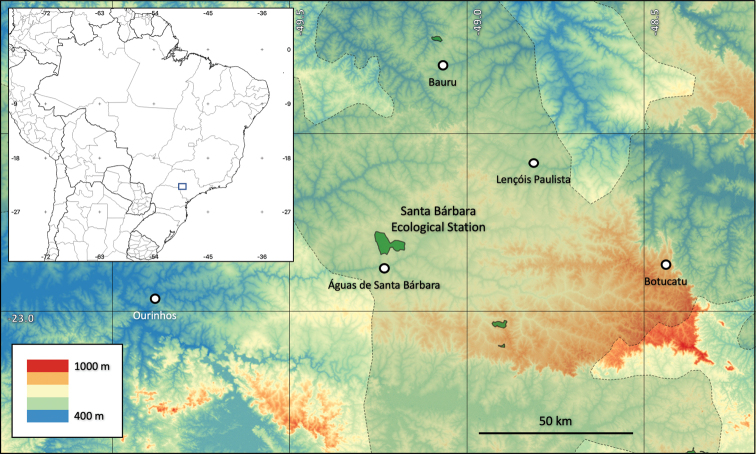
Topographic map of the region where the Santa Bárbara Ecological Station is located. Only the main cities of the region are shown. The Cerrado limits in this region are indicated by dashed lines. The green areas are strict conservation protected areas (PAs). Note that there are just four PAs in the Cerrado of this region.

**Figure 2.**
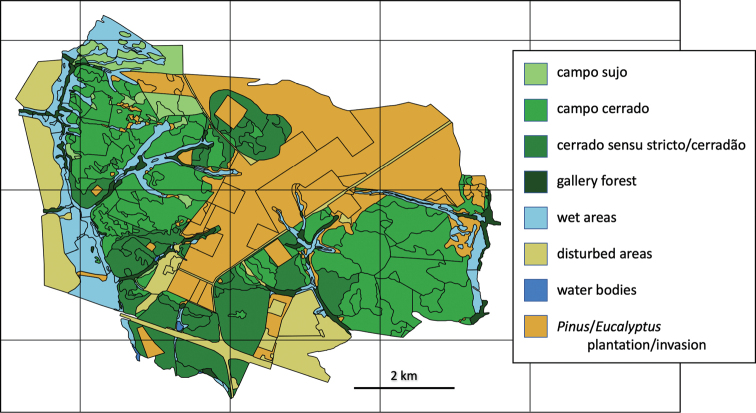
Land use types in the region of the Santa Bárbara Ecological Station.

This study was carried out at the Santa Bárbara Ecological Station (**SBES**), located in Águas de Santa Bárbara, State of São Paulo, Brazil (22°46' to 22°41'S and 49°16' to 49°10'W, elevation 600–680 m, Figure 1). SBES has a total area of 3,154 ha (Melo and Durigan 2011) and contains different Cerrado vegetation types, from open (such as campo sujo and campo cerrado) to forest (such as cerradão, a cerrado woodland, Figure 2), and some small areas with other types of forest vegetation (semi-deciduous seasonal forest, gallery forests, and pine and eucalyptus plantations), which were not the subject of this study.

It is estimated that the SBES harbours 4–9.5% of the total plant species present in the Cerrado (Meira-Neto et al. 2007). The climate is markedly seasonal, and the climate type is humid subtropical with a dry winter, type Cwa in the Köppen’s classification (Peel et al. 2007). The mean temperature is 17 °C in the coldest months and 24 °C in the warmest months. Maximum temperature is 35.2 °C and minimum, 3.4 °C. Frosts occur sporadically in Autumn and Winter. The annual rainfall is 1,010 to 2,051 mm (mean 1,454.2 mm) and there are marked dry (April to September, monthly mean 70.2 mm) and wet (October to March, monthly mean 172.1 mm) seasons (data for 1995–2014 at Manduri, state of São Paulo, 20.3 km from SBES; Centro integrado de informações agrometeorológicas, 2015). Most soils in the region are classified as red latosol, but grasslands usually occur on quartzene neossol and wet-fields on hydromorphic quartzene neossol; these neossols have high levels of sand and low fertility (Melo and Duringan 2011).

**Figure 3.**
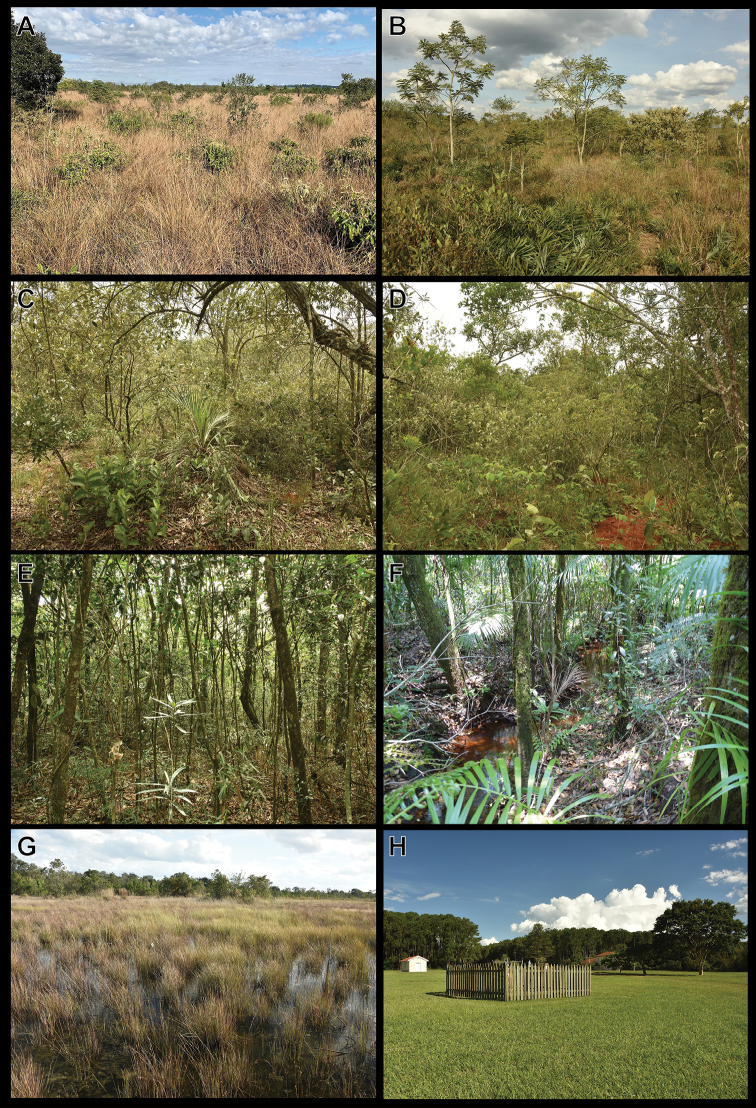
Natural vegetation types of Santa Bárbara Ecological Station **A** campo sujo (grassy scrubland) **B** campo cerrado (grassy scrubland with scattered trees) **C, D** cerrado sensu stricto (dense savanna) **E** cerradão (cerrado woodland) **F** gallery forest **G** wet field; and **H** disturbed area.

**Figure 4.**
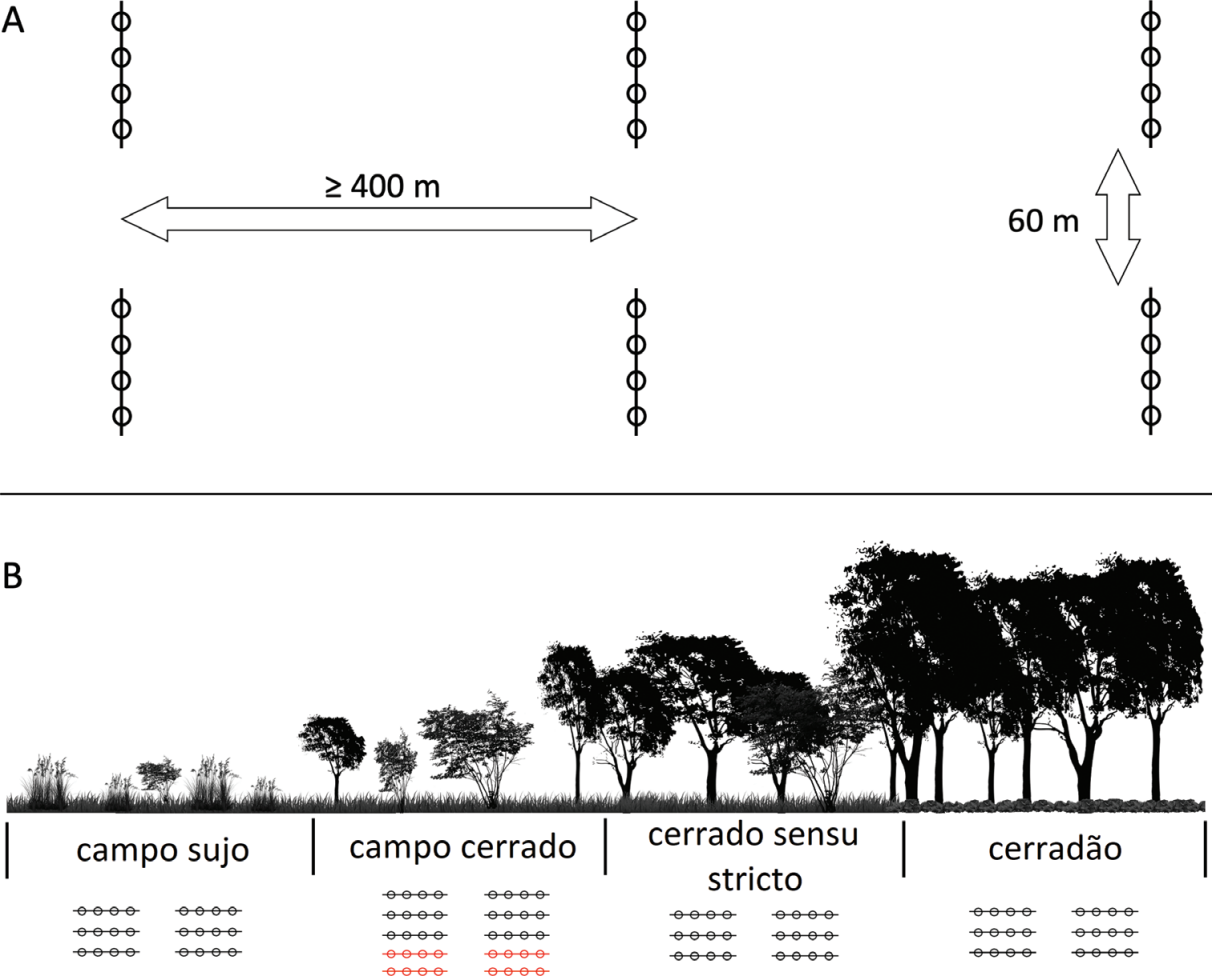
Schematic representation of the sampling design used in this study at the Santa Bárbara Ecological Station **A** representation of the distribution of three sampling units of pitfall traps (the sampling design in each vegetation type); each sampling unit was composed of two straight lines of drift fence (each of them 40 m long, 60 cm high), each line with four 100-L buckets 10 m apart from each other **B** representation of the sampling units used in each vegetation type; the red sampling units were fire treatments of a study on the effects of fire on the herpetofauna, results of which will be published elsewhere.

The samplings were performed periodically, for 10 days each month, from August 2016 to July 2018, for a total sample time of 240 days. Snakes were sampled with pitfall traps with drift fences (Greenberg et al. 1994; Cechin and Martins 2000), time-constrained searches (sensu Campbell and Christman 1982; Martins and Oliveira 1998), accidental encounters (Martins and Oliveira 1998), and assisted by the observations of local people (**OLP**; Martins and Nogueira 2012). Four main vegetation types were sampled with pitfall traps (**PT**) with drift fence: campo sujo, campo cerrado, cerrado sensu stricto, and cerradão (Figure 3). Our sampling design for pitfall traps included three sampling units per vegetation type, each sampling unit comprising two 40 m-long PT lines, located 60 m from each other, totalling 12 sampling units comprising a total of 24 lines and 96 buckets (Figure 4). Sampling units were located at least 400 m from each other. Each line had four 100 L plastic buckets connected by a 60 cm-high plastic fence, which was buried 10 cm below the soil surface and held upright by stakes. The buckets were perforated at the bottom to avoid accumulation of rainwater. A plastic plate (20 cm in diameter) and a piece of Styrofoam (20 × 20 cm) were placed in each bucket to provide moisture (plates were filled with water) and shelter for the captured animals. We also included individuals captured in four additional lines of PTs (16 buckets) located in two campo cerrado areas, which were part of a study on the effects of fire on the herpetofauna, results of which will be published elsewhere. Not all individuals recorded were collected.

Time-constrained searches (**TCS**; sensu Campbell and Christman 1982, Scott et al. 1989, Martins and Oliveira 1998) consisted of walking slowly in search of snakes in all visually accessible microenvironments. The sampling effort and the encounter rate were measured in person hours of visual search (Martins and Oliveira 1998). Sampling effort in TCS was not the same in all vegetation types. When performing TCS, we recorded the habitat (e.g., campo sujo, cerradão) and micro-habitats used by each snake (aquatic, arboreal, cryptozoic, fossorial, or terrestrial) and perch height (in case of arboreal species). To characterise micro-habitats, we used only information obtained during active searches; for individuals collected with PTs, only vegetation type (campo sujo, campo cerrado, cerrado sensu stricto, or cerradão) was recorded. Behavioural descriptions are based on observations made over short periods of time (ad libitum and sequence samplings; Altmann 1974). Defensive behaviours were recorded when individuals were observed in the field and when handled.

Accidental encounters (**AE**; Martins and Oliveira 1998), included all the specimens obtained by the researchers in activities other than the two-year regular sampling (August 2016–July 2018) with PTs and time-constrained searches. Accidental encounters occurred from June 2015 to December 2020 and were included in our database. OLP consisted of additional records made by people who live and/or work at the SBES, during their daily activities. When an animal was observed by these observers, they would photograph or warn researchers and we would go to the place to carry out the capture. At no time was the death of animals encouraged or requested (cf. Martins and Nogueira 2012).

The sampling efficiency of each method (AE, OLP, PT, and TCS) was evaluated through rarefaction curves of individuals with 5,000 randomisations (Sanders 1968; Gotelli and Colwell 2001), using number of individuals as a unit of sampling effort (Suppl. material 1). Dominance and Shannon diversity indices (H’) were also calculated in order to simultaneously evaluate snake diversity captured by each method (Suppl. material 1). Additionally, richness estimates were made for each method, using the First Order Jackknife estimator, in order to estimate how many species could still be found with each method (Suppl. material 1). This method (Jackknife I) estimates the total richness using the number of species that occur in only one (unique) sample (Burham and Overton 1979). These analyses were performed using the program EstimateS v. 8.20 (Colwell et al. 2012).

To describe the diet of each species, collected specimens (see below) were dissected through an incision in the ventral region. Food items were identified to the lowest possible taxonomic rank using taxonomic keys, identification guides, specimens deposited in scientific collections, and help from experts. Whenever the prey came from a snake captured in a PT, this information was included, given the possibility of the snake having ingested prey that had also fallen in the trap (Cechin and Martins 2000) but which is not part of the snake’s usual diet. To describe reproductive condition, we recorded the length of the largest ovarian follicle (**LOF**), the presence of egg or embryo, and the number of vitellogenic follicles (> 5 mm) per month of sampling.

The capture location of all animals sampled by the methods described above was georeferenced. In addition, all captured individuals were weighed and measured (snout-vent length; SVL). The average SVL (mean SVL) of each species was calculated based on adult individuals, which in turn were classified according to the SVL of the smallest reproductive female (containing ovarian follicles) or information from the literature. When adult individuals were not found, data from the literature were presented. New-born individuals were thus classified according to the presence of an evident umbilical scar. All captured individuals were released at the site of capture, except for voucher specimens. Owing to the importance of collecting voucher specimens for taxonomic identification and analysis of gastrointestinal and reproductive tracts to obtain useful natural history data, specimens and tissue samples were collected and preserved and these will later be deposited in the herpetological collections of the Museu de Zoologia da Universidade de São Paulo (**MZUSP**) and the Instituto Butantan (see Appendix 1). All collections were authorised by the Instituto Chico Mendes de Conservação da Biodiversidade (**ICMBio**; SISBIO permit #50658-1) and Comissão Técnico-Científica do Instituto Florestal (permit SMA #260108–011.518/2015). Specimens were euthanised by intracoelomic injection of lidocaine, fixed in 10% formaldehyde, and preserved in 70% ethanol (Foster 2012, AVMA 2020).

**Figure 5.**
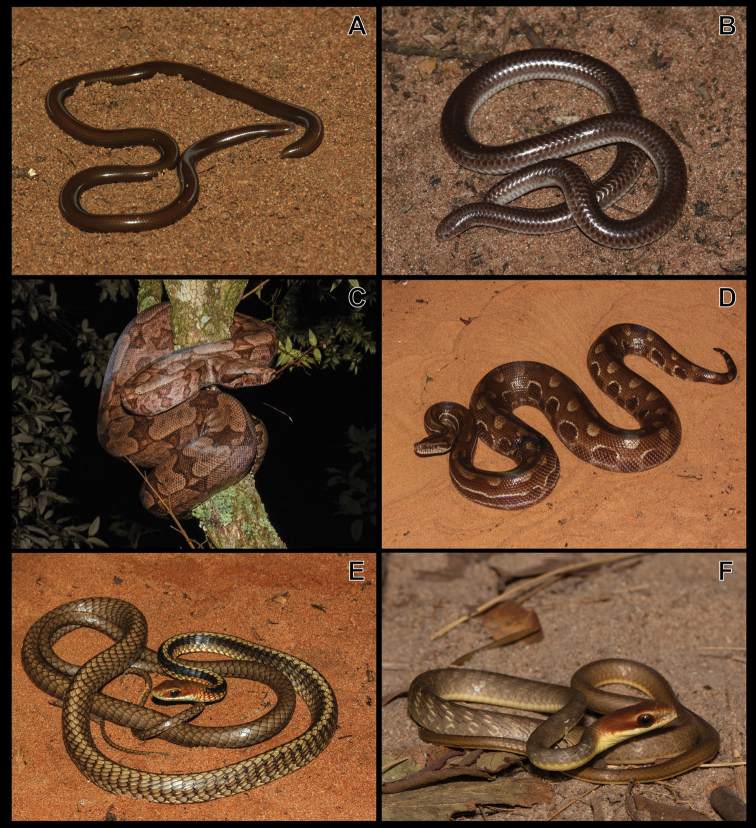
**A***Liotyphlopsternetzii***B***Trilepidakoppesi***C***Boaconstrictor***D***Epicratescrassus***E***Chironiusbrazili***F***C.quadricarinatus*.

**Figure 6.**
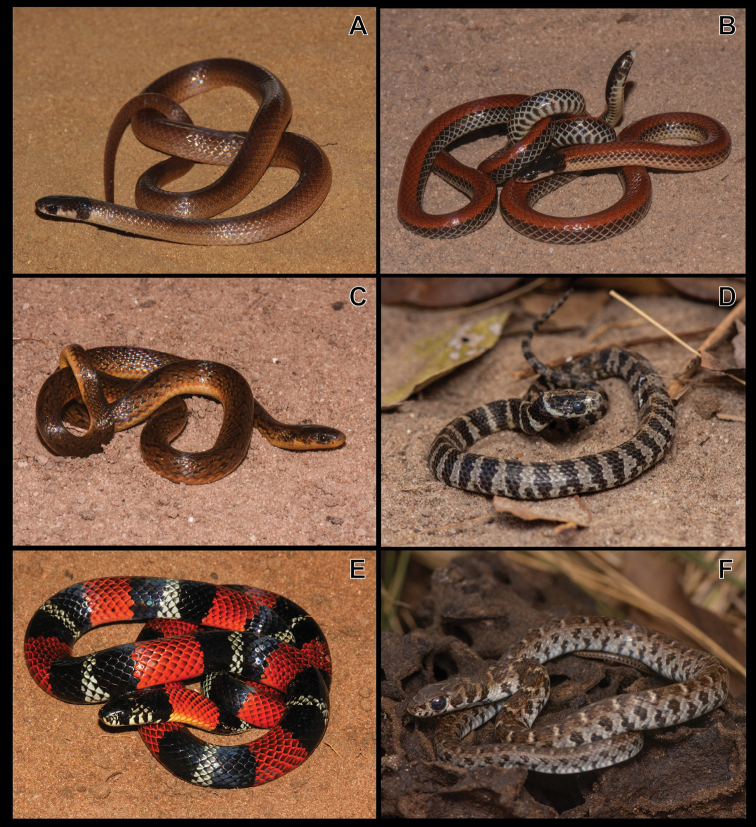
**A***Tantillamelanocephala***B***Apostolepisdimidiata***C***Atractuspantostictus***D***Dipsasmikanii***E***Erythrolamprusaesculapii***F***E.almadensis*.

**Figure 7.**
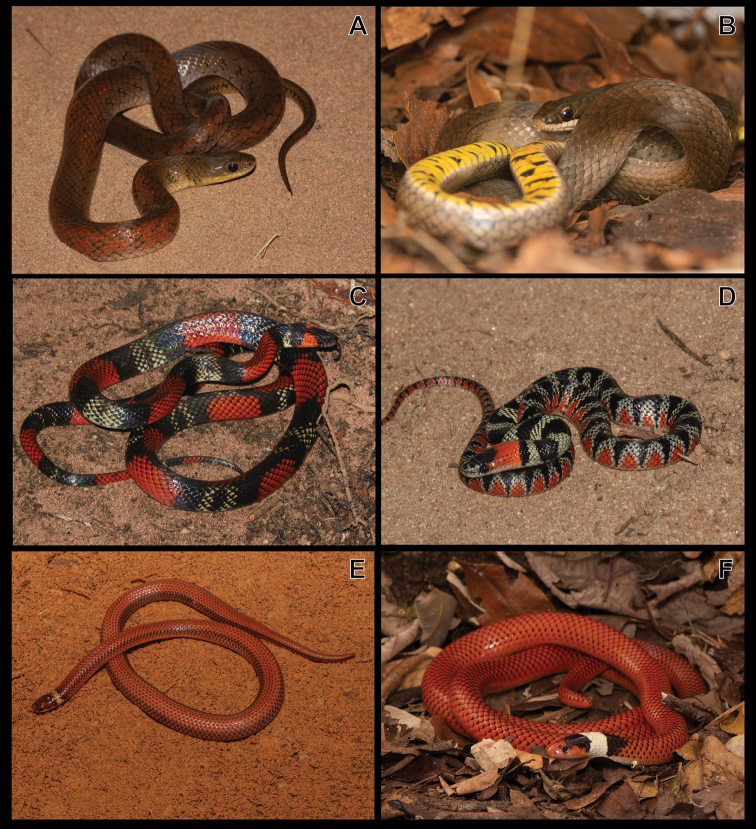
**A***Erythrolampruspoecilogyrus***B***E.reginae***C***Oxyrhophusguibei***D***O.rhombifer***E***Phalotrislativittatus***F***P.mertensi*.

**Figure 8.**
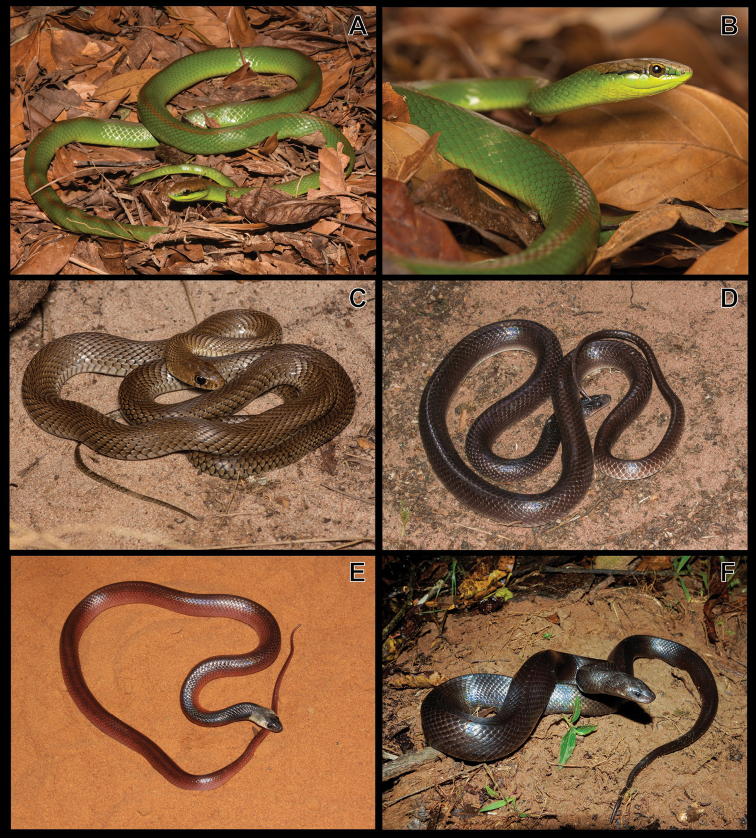
**A***Philodryasolfersii***B***P.olfersii* (detail of the head) **C***P.patagoniensis***D***Pseudoboanigra* (adult) **E***P.nigra* (juvenile) **F***Rachidelusbrazili*.

**Figure 9.**
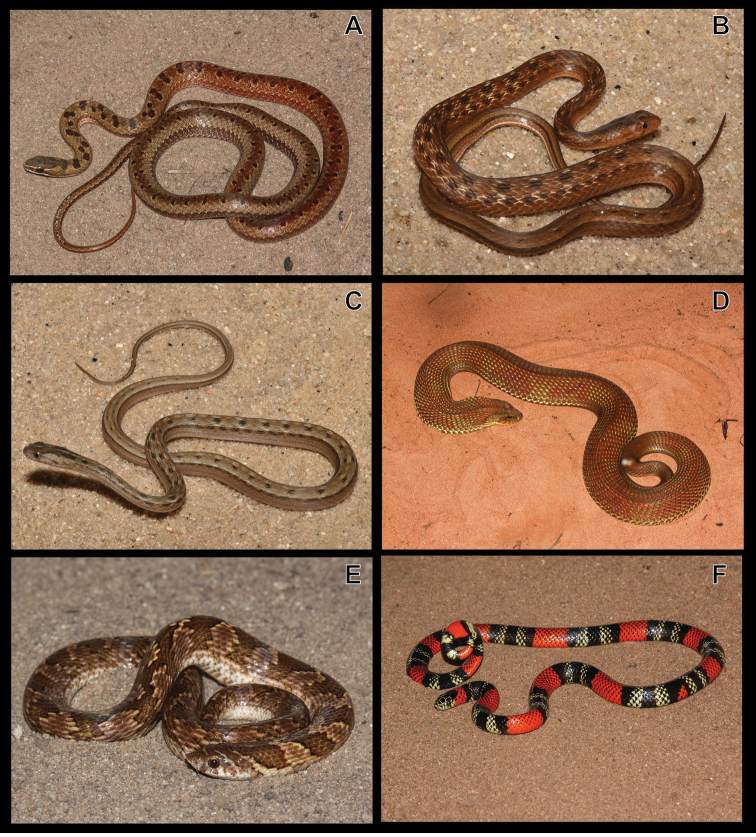
**A***Taeniophallusoccipitalis***B***Thamnodynasteshypoconia* (colour patterm 1) **C***T.hypoconia* (colour patterm 2) **D***Xenodonmerremi***E***X.nattereri***F***Micrurusfrontalis*.

**Figure 10.**
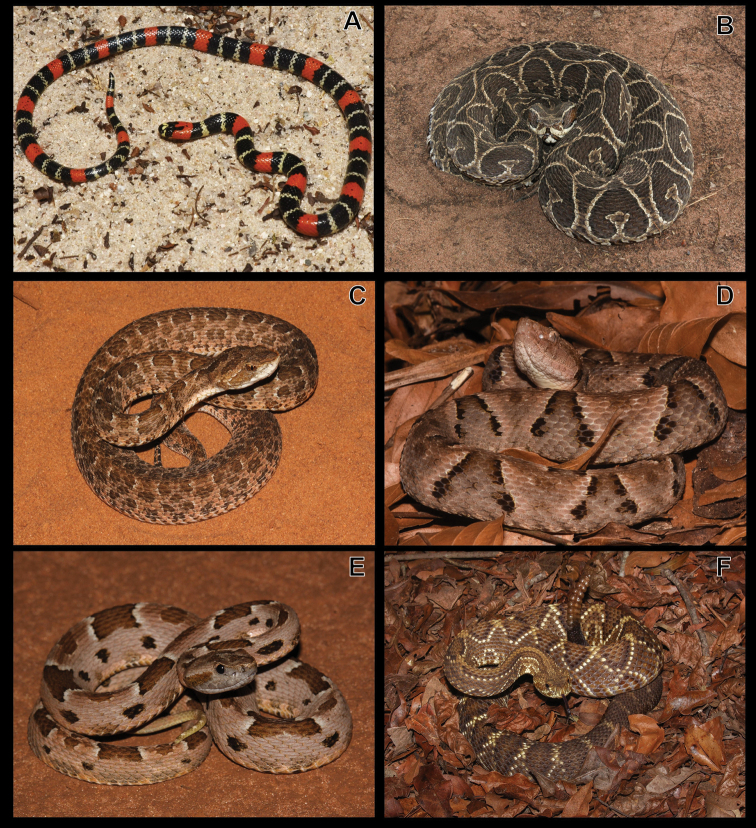
**A***Micruruslemniscatus***B***Bothropsalternatus***C***B.itapetiningae***D***B.moojeni***E***B.pauloensis***F***Crotalusdurissus*.

Besides the detailed natural history accounts for each species, we also provide a photographic guide (Figures 5–10) and an identification key to assist researchers and local people in identifying cerrado snakes from the SBES region.

## Results

With a sampling effort of 240 days of fieldwork, corresponding to 23,040 bucket-days and including 1248 person-hours of time-constrained search, we found 388 individuals (146 collected specimens; see Suppl. material 1) of 33 species of snakes (21 genera, seven families) at the SBES (Figures 5–10).

### General natural history patterns

Among all 33 species encountered using all described methods, approximately half of the species (18 species, 54%) used more often or were found exclusively in open (i.e., non-forest) vegetation types (campo sujo, campo cerrado or cerrado sensu stricto), while only *Boaconstrictor*, *Bothropsmoojeni*, *Erythrolamprusaesculapii*, *Erythrolamprusreginae*, *Phalotrismertensi*, and *Philodryasolfersi* were found most frequently (> 80% of the records for these species) in forest vegetation types (cerradão or gallery forests). Four species (*Apostolepisdimidiata*, *Crotalusdurissus*, *Pseudoboanigra*, and *Xenodonmerremi*) were considered habitat generalists (based also in the literature data), using both open and forest vegetation types (Table 1). Half of the species showed both diurnal and nocturnal activity (Table 1). The most consumed prey (based on the number of species that consume the prey, even occasionally, according to this study and the literature) were: lizards (42.4% of species), followed by amphibians (39.4%) and mammals (36.4%) (Tables 1, 2). Virtually all species mainly use the terrestrial micro-habitat (Table 1).

**Table 1.** Natural history summary of snakes found at Santa Bárbara Ecological Station, based on both data from this study and literature data. ACTIVITY = daily activity (D = diurnal; N = nocturnal); DIET (AN = anuran; AM = amphisbaenian; BE = bird eggs; BI = birds; FI = fish; IN = invertebrate; LI = lizard; LE = lizard eggs; MA = mammal; MO = mollusc; SN = snake); HABITAT = habitats used in the study area (CC = campo cerrado; CS = campo sujo; CD = cerradão; DA = disturbed area; GF = gallery forest; SS = cerrado sensu stricto; WF = wet field); MICRO-HABITAT (AQ = aquatic; AR = arboreal; CR = cryptozoic; FO = fossorial; TE = terrestrial). Uppercase letters represent the main resources and habits used according to the present study and the literature, while lowercase letters denote those used only occasionally (< 20% of the records in this study and/or rarely documented in the literature). Letters in parentheses indicate data obtained only from the literature and which were not observed in the present study, while an asterisk indicates that the observation is exclusive to the present study. Predominantly fossorial habits were inferred mainly from data in the literature and snake morphology (small eyes, flat skull, fused head scales and relatively short tail; Greene 1997).

Taxon	Habitat	Micro-habitat	Activity	Diet
** ANOMALEPIDIDAE **				
* Liotyphlops ternetzii *	CC	(FO)	(N, D)	(IN)
** LEPTOTYPHLOPIDAE **				
* Trilepida koppesi *	CC, cd, CS, SS	(FO), TE	N, (d)	IN
** BOIDAE **				
* Boa constrictor *	CD, DA, gf	(AR), TE	D, (N)	BI, (li), MA
* Epicrates crassus *	CC, CS, CD	ar, TE	d, N	(BI), MA
** COLUBRIDAE **				
* Chironius brazili *	DA	(AR), TE	D	(AN)
* Chironius quadricarinatus *	SS	(AR), TE	D	(AN)
* Tantilla melanocephala *	CC, CS	(FO), TE	(D, N)	IN
** DIPSADIDAE **				
* Apostolepis dimidiata *	CC, CD, cs, ss	cr, (FO), TE	(N)	(AM), li*, (SN)
* Atractus pantostictus *	CC, CS, GF	(CR), (FO), TE	(D), N	IN, (li)
* Dipsas mikanii *	DA	TE	D, (N)	(MO)
* Erythrolamprus aesculapii *	CD, da	CR, TE	D	SN
* Erythrolamprus almadensis *	CS	(AQ), TE	(D)	(AN, FI)
* Erythrolamprus poecilogyrus *	CC, CS, DA, SS	TE	D, N	(AN)
* Erythrolamprus reginae *	CD	(AQ), TE	(D), (N)	AN, LI
* Oxyrhopus guibei *	CC, CS, da, ss, wf	TE	N	ma, LI
* Oxyrhopus rhombifer *	CS	TE	(N)	(MA, LI, sn)
* Phalotris lativittatus *	da, CC, cd, SS	(FO), TE	N	AM, SN
* Phalotris mertensi *	(CS), CD	(FO), te	D, (N)	(AM)
* Philodryas olfersi *	CD	(AR), TE	D	AN, MA
* Philodryas patagoniensis *	CS, (DA, SS)	TE	(D)	(AN, bi, LI, ma, sn)
* Pseudoboa nigra *	CS, GF	TE	N	(am), LI, (ma, sn)
* Rhachidelus brazili *	CS, CC, (da)	TE	N	(BE), bi
* Taeniophallus occipitalis *	CS, CC, SS	(CR), TE	D, N	(AN), LI
* Thamnodynastes hypoconia *	cs, WF	(AR), TE	N	(AN)
* Xenodon merremi *	CD, DA	cr, TE	D	(AN)
* Xenodon nattereri *	CS, CC	(FO), TE	(D)	(LI,sn), LE
** ELAPIDAE **				
* Micrurus frontalis *	CC	(FO), TE	(D, N)	(AM, LI, SN)
* Micrurus lemniscatus *	CC	(FO), TE	(D, N)	(AM, FI, SN)
** VIPERIDAE **				
* Bothrops alternatus *	CS, cc, da	TE	D, N	MA
* Bothrops itapetiningae *	CS, CC	TE	D, N	(an, bi, LI, MA)
* Bothrops moojeni *	cc, cd, da, GF, ss	(ar), TE	d, N	AN, MA
* Bothrops pauloensis *	CC, cd, cs, da	TE	d, N	an, (bi, LI, MA, sn)
* Crotalus durissus *	DA, cc, CD, cs, gf, ss	TE	D, N	(li), MA

**Table 2.** Occurrence (number of snakes which presented a given gut content) and total number of prey items consumed by snakes from Santa Bárbara Ecological Station, SP, Brazil. Asterisks indicate individuals captured in pitfall traps.

Taxon	Gut contents	Occurrence	Number of prey items
** Leptotyphlopidae **			
* Trilepida koppesi *	Isoptera	3*	12*
	insect eggs	1*	9*
	Formicidae pupae	3*	12*
** Boidae **			
* Boa constrictor *	feathers *Didelphisalbiventris*	1	1
		1	1
	* Guira guira *	1	1
* Epicrates crassus *	mammal	1	1
** Colubridae **			
* Tantilla melanocephala *	Chilopoda	1; 3*	1; 3*
** Dipsadidae **			
* Apostolepis dimidiata *	Teiidae skin fragments	1*	1*
* Atractus pantostictus *	Oligochaeta	1; 1*	1; 1*
* Erythrolamprus aesculapii *	snake scale	1	1
* Erythrolamprus reginae *	lizard scale	1*	1*
	*Physalaemus* sp.	1*	1*
* Oxyrhopus guibei *	* Aspronema dorsivittatum *	4*	4*
	Teiidae scale	1*	1*
	Gymnophtalmidae skin fragments	1*	1*
	rodent	1	1
* Phalotris lativittatus *	Amphisbaenian fragments	1	1
	*Trilepidakoppesi* scales	1*	1*
* Philodryas olfersii *	* Oligoryzomys nigripes *	1	1
	* Scinax fuscovarius *	1	1
* Pseudoboa nigra *	*Ameiva* sp. scales	1	1
* Rhachidelus brazili *	bird bones and feathers	1	1
* Taeniophallus occipitalis *	Teiidae fragments	1; 1*	1; 1*
* Xenodon nattereri *	lizard eggs	1	2
** Viperidae **			
* Bothrops alternatus *	* Clyomys laticeps *	1	1
* Bothrops moojeni *	*Leptodactylus* sp.	1	1
	rodent	1	1
* Bothrops pauloensis *	*Leptodactylus* sp.	1	1
* Crotalus durissus *	* Calomys tener *	1	1
	mammal	5	5

As expected, the different lineages in the community showed some differences regarding their natural history. The snakes of the family Dipsadidae (most represented in the community) showed similar habits within tribes, where those belonging to the tribe Elapomorphini (*A.dimidiata*, *P.lativittatus*, and *P.mertensi*) used forests (although *P.lativittatus* uses primarily open areas), exhibit (at least in part) nocturnal activity (*A.dimidiata* and *P.lativittatus* are nocturnal species and although *P.mertensi* has been found thermoregulating during the day, this species probably is also primarily nocturnal) and consume elongated fossorial vertebrates (caecilians, amphisbaenians, and other snakes). Among the species of the tribe Pseudoboini (*O.guibei*, *O.rhombifer*, *P.nigra*, and *R.brazili*), except for *P.nigra*, all were found exclusively in open areas, active during the night and include primarily mammals and/or lizards in their diets (except for *R.brazili*, which is specialised in birds and their eggs). In the tribe Xenodontini, most species showed primarily daytime activity and preyed mostly on anurans, all of which are primarily terrestrial and/or cryptozoic.

The vipers of the genus *Bothrops* differed in terms of habitat use according to the respective clade within the genus (see Carrasco et al. 2011). The species of the *B.alternatus* group (*B.itapetiningae* and *B.alternatus*) strictly occupied the most open areas of the reserve (campo sujo and campo cerrado), the representative of the *B.neuwiedi* group (*B.pauloensis*) was also more frequent in open areas, although it was not as restricted to these habitats (it was found also in cerrado sensu stricto and in cerradão), whereas the representative of the *B.atrox* group (*B.moojeni*) was associated with forests (gallery forests) and wet fields. The rattlesnake, *C.durissus*, was found in all habitats.

We obtained 48 gut contents from 138 individuals (specimens dissected or live individuals that regurgitated or defecated prey remains; Table 2). Among the specimens examined, 22 females belonging to 18 species contained primary and secondary ovarian follicles. The largest number of females containing vitellogenic follicles (> 5 mm) occurred during the wet season (October–March; Table 3) and we had a higher number of records of new-borns during the dry season (April to September) (Table 3), with temporal overlapping of the different families (Anomalepididae, Leptotyphlopidae, Colubridae, Dipsadidae, and Viperidae) regarding the presence of ovarian follicles and new-borns.

**Table 3.** Temporal distribution of the number of ovarian follicles (F) and new-borns (NB) of different species found at Santa Bárbara Ecological Station between August 2016 and July 2018. Numbers in parentheses represent the length of the largest ovarian follicle (mm) and asterisks indicate a secondary or vitellogenic ovarian follicle (> 5 mm).

Species	Jan	Feb	Mar	Apr	May	Jun	Jul	Aug	Sep	Oct	Nov	Dec
	wet season			dry season						wet season		
* Liotyphlops ternetzii *						1NB						
* Trilepida koppesi *		19F(3.5)								15-48F(21.6)*		
* Chironius brazili *				21F(5.2)*								
* Tantilla melanocephala *								8F(3.8)	11F(6.5)*			
* Apostolepis dimidiata *			6F(5)	1NB								1F(24)*
* Atractus pantostictus *		7F(9.7)*	37F(9)*									
* Dipsas mikanii *						1NB						
* Erythrolamprus aesculapii *	5F(7.6)*											
* Erythrolamprus almadensis *							1NB					
* Erythrolamprus reginae *		6F(16.5)*										
* Oxyrhopus guibei *										7F(27.7)*		
* Oxyrhopus rhombifer *			1NB									
* Phalotris lativittatus *		1F(7.4)*										
* Philodryas olfersii *			22F(11)*									
* Taeniophallus occipitalis *		1NB		1NB					17F(8.7)*			
* Xenodon merremii *		26F(7.2)*										
* Xenodon nattereri *										8F(3.8)		
* Bothrops alternatus *								14F(13.7)*				
* Bothrops itapetiningae *											7F(3.9)	
* Bothrops moojeni *									1NB	28F(5.2)*		
* Bothrops pauloensis *	18F(4.5); 1NB	1NB				1NB		11F(6)*; 15F(3.6)				
* Crotalus durissus *				1NB	1NB			8F(4.3)				

### Keys to families and species of snakes from Santa Bárbara Ecological Station, SP, Brazil

**Table:** 

1	Ventral and dorsal scales of the same size	**Anomalepididae and Leptotyphlopidae**
–	Ventral and dorsal scales of distinct sizes	**2**
2	Loreal pit present	** Viperidae **
–	Loreal pit absent	**3**
3	Proteroglyphous dentition	** Elapidae **
–	Other dentition types	**4**
4	Aglyphous dentition; cephalic scutes small, undifferentiated; > 30 dorsal scale rows	** Boidae **
–	Solenoglyphous dentition; cephalic scutes large, with different forms; fewer than 30 dorsal scale rows	**Colubridae and Dipsadidae**

**Species of the families Anomalepididae and Leptotyphlopidae**

**Table:** 

1	Ocular scale in contact with mouth	*** Trilepida koppesi ***
–	Ocular scale not in contact with mouth	** Liotyphlops cf. ternetzii **

**Species of the family Viperidae**

**Table:** 

1	Presence of a rattle or a button at the tip of the tail; some large plates on the dorsum of the head	*** Crotalus durissus ***
–	No rattle or button at the tip of the tail; no large plates on the dorsum of the head	**2**
2	Prelacunal scale fused with the second supralabial scale	*** Bothrops moojeni ***
–	Prelacunal scale not fused with the second supralabial scale	**3**
3	Postocular stripe inserted in the inferior region of the ocular orbit	**4**
–	Postocular stripe not inserted in the inferior region of the ocular orbit	*** Bothrops pauloensis ***
4	Colour pattern with trapezoidal dark marks; > 11 supralabial scales	*** Bothrops alternatus ***
–	Colour pattern not as above; < 11 supralabial scales	*** Bothrops itapetiningae ***

**Species of the family Elapidae**

**Table:** 

1	Parietals completely black	*** Micrurus frontalis ***
–	Posterior portion of parietals red	*** Micrurus lemniscatus ***

**Species of the family Boidae**

**Table:** 

1	Supralabials in contact with eye	*** Epicrates crassus ***
–	Suprelabials not in contact with eye	*** Boa constrictor ***

**Species of the families Colubridae and Dipsadidae**

**Table:** 

1	Even number of dorsal scale rows	**2**
–	Odd number of dorsal scale rows	**3**
2	Anterior third of the body blackish, with a yellowish vertebral stripe	*** Chironius flavolineatus ***
–	Anterior third of the body brown or light grey, without a vertebral stripe	*** Chironius quadricarinatus ***
3	15 or fewer dorsal scale rows at midbody	**4**
–	More than 15 dorsal scale rows at midbody	**11**
4	13 dorsal scale rows at midbody	*** Philodryas agassizii ***
–	15 dorsal scale rows at midbody	**5**
5	A single prefrontal scale	**6**
–	A pair of prefrontal scales	**7**
6	Presence of dark longitudinal stripes along the flanks; snout slightly pointed, with prominent rostral scute	*** Phalotris lativittatus ***
–	No dark longitudinal stripes along the flanks; snout rounded, rostral scute not prominent	*** Phalotris mertensi ***
7	Internasal scutes absent	*** Apostolepis dimidiata ***
–	Internasal scutes present	**8**
8	Coral colour pattern of alternating black, yellow/white, and red bands	*** Erythrolamprus aesculapii ***
–	Colour pattern not as above	**9**
9	Black bands throughout the dorsum; dark oral and cloacal mucosae	*** Dipsas mikanii ***
–	Colour pattern not as above; light oral and cloacal mucosae	**10**
10	Head brown to greyish, often with a pair of light ocelli on the parietal scutes; yellow venter; < 175 ventral scales	** Taeniophallus gr. occipitalis **
–	Head black, without a pair of light ocelli on the parietal scutes; cream to whitish venter; > 175 ventral scales	*** Tantilla melanocephala ***
11	More than 19 dorsal scales at midbody	*** Rhachidelus brazili ***
–	19 or fewer dorsal scales at midbody	**12**
12	17 dorsal scales at midbody	**13**
–	19 dorsal scales at midbody	**14**
13	Venter yellow with black marks; dorsal scales with reduction in number towards cloaca (17/17/15)	*** Erythrolamprus reginae ***
–	Venter uniformly cream; dorsal scales without reduction in number (17/17/17)	*** Atractus pantostictus ***
14	Anal plate entire	**15**
–	Anal plate divided	**17**
15	Dorsum and flanks black with or without white areas; juveniles with a white band on the dorsum of the head and a nuchal black and dorsum rose-red; subcaudals entire	*** Pseudoboa nigra ***
–	Coral colour pattern (alternated black, yellow/white, and red bands); subcaudals divided	**16**
16	Coral colour pattern in trios	*** Oxyrhopus guibei ***
–	Coral colour pattern not in trios	*** Oxyrhopus rhombifer ***
17	Dorsal scale rows in 19/19/15	**18**
–	Dorsal scale rows in 19/19/17	**21**
18	Keeled dorsal scales	*** Thamnodynastes hypoconia ***
–	Smooth dorsal scales	**19**
19	Green dorsum, brown head and vertebral stripe	*** Philodryas olfersii ***
–	Colour pattern not as above	**20**
20	7 supralabials, third and fourth in contact with the ocular orbit	*** Philodryas patagoniensis ***
–	8 supralabials, fourth and fifth in contact with the ocular orbit	*** Erythrolamprus poecilogyrus ***
21	Snout pointed, with rostral scute upturned and keeled	*** Xenodon nattereri ***
–	Snout round, without rostral scute upturned and keeled	**22**
22	Venter orange-red from the 2/3 of the body onward; 8 supralabials, fourth and fifth in contact with the ocular orbit	*** Erythrolamprus almadensis ***
–	Venter cream; 7 supralabials, third and fourth in contact with the ocular orbit	*** Xenodon merremii ***

### Natural history accounts

#### Anomalepididae Taylor, 1939

***Liotyphlopsternetzii* (Boulenger, 1896)**

Figure 5A

It is a small aglyphous species (mean SVL = 239 mm; range 93–319 mm; N = 30; França et al. 2008). It was found only in campo cerrado (N = 3), always after heavy rains. As all individuals were captured in PT, information on micro-habitat use or daily activity was not obtained. The available information indicates that the species is fossorial and both diurnal and nocturnal (França and Araújo 2006; Marques et al. 2015) and that it feeds on ants and termites (França and Araújo 2006). The largest individuals (SVL = 189 and 211 mm, respectively) were captured in January and February, respectively, and the third individual, a newborn, in June. It can lay two to seven eggs during the wet season (November–December: Achaval and Olmos 1997). A new-born was found (CRC = 90 mm) in the dry season (June) in this study. When handled, it pressed the sharp tip of the tail against the captor’s hand, a behaviour also observed by Marques et al. (2015).

#### Leptotyphlopidae Stejneger, 1892

***Trilepidakoppesi* (Amaral, 1955)**

Figure 5B

It is a small aglyphous species (mean SVL = 245 mm; range 198–323 mm; mature males, SVL ≥ 198 mm, and mature females, SVL ≥ 225 mm; N = 83). This species was found more frequently in open areas, such as campo sujo (N = 51) and campo cerrado (N = 62), but also in cerrado sensu stricto (N = 21) and in cerradão (N = 1). Almost all observations through TCS (only one through AE) of active individuals (moving) were made at night, between 07:00 and 09:00 PM (N = 14). Among eight individuals examined, four had stomach contents, three ingested termites (MRCM 290, 355, 383) and pupae of ants (MRCM 290, 328, 383) and one, insect eggs (MRCM 290). It is known to be fossorial and both diurnal and nocturnal (França and Araújo 2006; Sawaya et al. 2008) and feeds exclusively on invertebrates such as larvae and pupae of ants and occasionally adult ants, termite larvae and beetles (França and Araújo 2006, Sawaya et al. 2008). Virtually all captured individuals were found during the wet season (October to March), with the largest number of individuals being observed in October 2016 (N = 36) and the smallest in April 2017 (N = 1). Most females with ovarian follicles (LOF = 21.6 mm; MRCM 290, 292, 312, 318, 355, 381, 382, 383) were also found in October and a single female with follicles was found in February (LOF = 3.5 mm; MRCM 457). The available information indicates that it is oviparous and has relatively low fertility (5–7 eggs), with egg laying probably occurring in the middle of the wet season, starting in December (Sawaya et al. 2008). When handled, it can press the sharp tip of the tail against the captor’s hand (N = 11; MRCM 290, 308, 309, 323), as already described in the literature (Marques et al. 2015), it also produces cloacal discharge (N = 27; MRCM 290, 309, 314, 323) or bite (N = 1) (Table 4). It can also twist its body when handled (Sawaya et al. 2008).

**Table 4.** Defensive tactics of snakes from the Santa Bárbara Ecological Station, SP, Brazil. BI = bite; CB = compress body while raising head; CD = cloacal discharge; DF death feigning; EM = perform erratic movements; FB = flatten body; FBL = flatten body laterally; HH = hide head; OM = open mouth; PT = press the sharp tip of the tail against the captor’s hand; ST = strike; TB = twist the body; TD = tail display; TH = triangulate head; SC = S-coil; VT = vibrate tail. The numbers indicate field observations, and an “X” indicates data from the literature (Martins et al. 2008; Sawaya et al. 2008; Marques et al. 2015; Fiorillo et al. 2020a).

Species	BI	CB	CD	DF	EM	FB	FBL	HH	OM	PT	ST	TB	TD	TH	SC	VT
* Liotyphlops ternetzii *										X						
* Trilepida koppesi *	1		27							11		X				
* Boa constrictor *	2								1		1					
* Epicrates crassus *			X					X			X		X			
* Chironius brazili *	1								1						1	
* Chironius quadricarinatus *	1		X				X				1				1	
* Tantilla melanocephalla *			X									X				
* Apostolepis dimidiata *								X		1						
* Atractus pantostictus *			X		X					1						
* Dipsas mikanii *		X	X		1			X						1		
* Erythrolamprus aesculapii *	2	X	1		1	3		X					3			
* Erythrolamprus almadensis *			X													
* Erythrolamprus poecilogyrus *			1			X						X				
* Erythrolamprus reginae *			2		1	2										
* Oxyrhopus guibei *			1		3			X				X	1			
* Oxyrhopus rhombifer *			X		X	X		X				X				
* Phalotris lativittatus *					X					X						
* Phalotris mertensi *					1				X	X						
* Philodryas olfersii *			X						X		X				X	
* Philodryas patagoniensis *	X		X			X						X		X		
* Pseudoboa nigra *			1		X			1								
* Rhachidelus brazili *			1		1	1								1		
* Taeniophallus occipitalis *			X	X		X						1				
* Thamnodynastes hypoconia *	2		X			X			X		X	X		X		
* Xenodon merremii *		1				2		1	1		2		X	1		
* Xenodon nattereri *						X		X					X			
* Micrurus frontalis *					1	X		1					1			
* Micrurus lemniscatus *					1	X		1					1		1	
* Bothrops alternatus *						2		2			3					2
* Bothrops itapetiningae *			X			1		X	X		1					2
* Bothrops moojeni *	1		X			X		1	X		3					4
* Bothrops pauloensis *						1		1			16	1				12
* Crotalus durissus *			2			X					5					6

#### Boidae Gray, 1825

***Boaconstrictor* Linnaeus, 1758**

Figure 5C

It is a large aglyphous species (mean SVL = 1,122 mm; range 935–1,332 mm; N = 6; this study). Four individuals were found on the edge of forests or in forest areas (two occasionally on the edge of cerradão, one OLP on the edge of gallery forests, and the other OLP on a *Eucalyptus* plantation with surrounding forest) and five were captured in a disturbed area close to the SBES headquarters, also OLP. All individuals were moving on the ground during the day (between 07:00 AM and 04:00 PM). The available information indicates that the species is semi-arboreal and both diurnal and nocturnal (Martins and Oliveira 1998; Bernarde and Abe 2006; França and Araújo 2006). One adult male defecated bird feathers and another adult male (MRCM 386) regurgitated a freshly ingested marsupial (*Didelphisalbiventris*). A third individual (not sexed) was observed preying on a Guira Cuckoo (*Guiraguira*) on the ground, in a disturbed area near to the SBES headquarters at ca. 03:00 PM. The available information indicates that the species feeds mainly on mammals and birds and occasionally lizards (Martins and Oliveira 1998; Bernarde and Abe 2006; Marques et al. 2015). The species was observed throughout the year, but mostly during the wet season (five individuals between October and March). It has a seasonal reproductive cycle, with vitellogenesis in the summer (January–February) and it reaches sexual maturity with at least 1300 mm SVL in females and 1100 mm in males (Pizzatto and Marques 2007). Pregnancy lasts 4–6 months (Pizzatto and Marques 2007) and it can reproduce asexually by parthenogenesis (Bertona and Chiaraviglio 2003; Booth et al. 2011). When handled, it can bite (N = 2; MRCM 386), strike (N = 1), or open its mouth wide (N = 1) (Table 4), as already described in the literature (Sawaya et al. 2008; Marques et al. 2015).

***Epicratescrassus* Cope, 1862**

Figure 5D

It is a large aglyphous species (mean SVL = 893 mm; range 704–1000.5 mm; N = 4; this study). One individual was found in the campo sujo (AE), three in the campo cerrado (AE = 2, TCS = 1; MRCM 395) and one in the edge of a forest (TCS; MRCM 359). Three of them were moving during the night and one during the day. An individual was found perched on the drift fence of one of the PTs (MRCM 395). One of the specimens examined contained hair in its stomach (MRCM 359). The available information indicates that the species is both diurnal and nocturnal, has terrestrial habits, and feeds mainly on birds and mammals (Sawaya et al. 2008; Cassimiro et al. 2010; Marques et al. 2015). All individuals were observed during the wet season, from November to March. It presents a seasonal reproductive cycle, producing 8–22 hatchlings per litter, and reaches sexual maturity with at least 970 mm SVL in females and 870 mm in males (Pizzatto and Marques 2007). When handled, it can strike, hide its head, perform tail display, or produce cloacal discharge (Sawaya et al. 2008; Marques et al. 2015; Table 4).

#### Colubridae (Ooppel, 1811)

***Chironiusbrazili* Hamdan & Fernandes, 2015**

Figure 5E

It is a large aglyphous species (mean SVL = 893 mm; range 256–995 mm; N = 37; Hamdan and Fernandes 2015). The only individual found was observed by OLP (MRCM 482), in a disturbed area (open flooded area adjacent to a pine forest), at ca. 03:00 PM. The species occupies areas in the southern portion of the Cerrado in contact with the Atlantic Forest (Nogueira et al. 2019) and, apparently, forest habitats such as gallery forests (Hamdan and Fernandes 2015). The individual found was a female observed in April (late wet season) and had 21 ovarian follicles (LOF = 5.2 mm). When handled, the species can open the mouth wide (showing the oral mucosa), bite or raise its head, and form an S-coil with the anterior part of its body (N = 1) (Table 4).

***Chironiusquadricarinatus* (Boie, 1827)**

Figure 5F

Large aglyphous species (mean SVL = 633; range 504–808 mm; N = 108; Pinto et al. 2010). The single individual found AE (MRCM 1275), in cerrado sensu stricto, moving on the ground at 11:00 AM. The available information indicates that it is semi-arboreal and diurnal (Dixon et al. 1993, França and Araújo 2006), can occur in disturbed areas (Carvalho and Nogueira 1998) and feeds mainly on frogs (Dixon et al. 1993; França and Araújo 2006; Pinto et al. 2008). The individual found was a juvenile (SVL = 250 mm) and was sampled in April (onset of dry season). This species has a continuous reproductive cycle and fecundity varies from three to nine eggs (Dixon et al. 1993; Pinto 2006). It reaches sexual maturity with at least 514 mm SVL in females and 504 mm in males (Pinto et al. 2010). When handled, it can bite, strike, or form an S-coil with the anterior part of its body (N = 1) (Table 4). The available information indicates that it can also flatten the body laterally and produce cloacal discharge (Marques et al. 2015; Table 4).

***Tantillamelanocephala* (Linnaeus, 1758)**

Figure 6A

It is a small opisthoglyphous species (range 181–296 mm; N = 146; Santos-Costa et al. 2006). In the SBES, it was observed only in open areas, campo sujo (AE = 1, PT = 5; MRCM 274, 291, 461, 514, 544) and campo cerrado (AE = 2, PT = 2; MRCM 345, 1298), all captured through PT. One individual was found at night under a fallen log in a wet area (AE). The available information indicates that the species is fossorial, cryptozoic, and both diurnal and nocturnal (Martins and Oliveira 1998; Martins et al. 2008; Marques et al. 2015). Among four individuals examined, all had remains of centipedes in the gut (MRCM 274, 461, 544, 1298). It is specialised in feeding on centipedes (Cunha and Nascimento 1978; Marques and Puorto 1998; Marques et al. 2015). Among the collected females, two (MRCM 274, 1298) had ovarian follicles (11 follicles and LOF = 6.5 mm, and eight follicles and LOF = 3.8 mm respectively), during the dry season (August and September). According to the literature, their different populations may present a seasonal or continuous reproduction (Marques and Puorto 1998; Santos-Costa et al. 2006). Its fecundity varies from one to three eggs (Fitch 1970; Dixon and Soini 1986; Sawaya et al. 2008); in the Amazon rain forest, males reach sexual maturity with at least 181 mm SVL and females with 189 mm (Santos-Costa et al. 2006). When handled, it can twist the body and produce cloacal discharge (Sawaya et al. 2008; Marques et al. 2015; Table 4).

#### Dipsadidae Bonaparte 1838

***Apostolepisdimidiata* (Jan, 1862)**

Figure 6B

It is a medium-sized opisthoglyphous species (mean SVL = 387.8 mm; range 196–634 mm; N = 46; Sawaya et al. 2008). It was found in all vegetation types: three in campo sujo (MRCM 545, 1702), six in campo cerrado (MRCM 512, 524, 1256, 1802), one in a cerrado sensu stricto and five in a cerradão (MRCM 387, 463, 483, 541). One of the individuals was AE buried under the leaf litter during the day (MRCM 387). Sawaya et al. (2008) also found individuals mostly in open areas, although also in disturbed areas outside Itirapina Ecological Station. The available information indicates that it is predominantly nocturnal and has fossorial habits (Sawaya et al. 2008; Marques et al. 2015). Among six specimens examined, only one had stomach contents (skin fragments from a lizard of the family Teiidae; this study; MRCM 545). It feeds on amphisbaenians (Sawaya et al. 2008) and possibly on other elongate vertebrates (Marques et al. 2009). At the SBES, most individuals were observed during the wet season or late in the dry season (late September); a new-born was found in April. Among the females examined, one (collected in March) had six ovarian follicles (LOF = 5 mm; MRCM 1296) and the other (collected in December) had a follicle that was probably about to enter its uterus (24 mm; MRCM 387). When handled, the species pressed the sharp tip of the tail against the captor’s hand (N = 1; MRCM 463) as already described in the literature (Marques et al. 2015; Table 4). The available information indicates that it can also hide its head (Sawaya et al. 2008).

***Atractuspantostictus* Fernandes & Puorto, 1993**

Figure 6C

It is a small aglyphous species (mean SVL = 269 mm; range 122–420 mm; N = 10; França et al. 2008). Most of the individuals were observed in the most open areas of the SBES, in campo sujo (PT = 2; MRCM 352, 427) and campo cerrado (PT = 2; MRCM 389, 1100). However, an individual was observed (during TCS) moving among the grass at the edge of a gallery forest (MRCM 11). Only the latter individual was observed when active, during the night (07:30 PM). The available information indicates that the species is both diurnal and nocturnal (Martins et al. 2008), cryptozoic (França and Araújo 2006), and fossorial (Rocha-Barbosa and Moraes-e-Silva 2009). Among the four individuals examined, two had earthworm setae in their hindgut (MRCM 11, 427). The species is known to eat worms (Sawaya et al. 2008; Marques et al. 2009; Marques et al. 2015) and there is a record for lizard, with scales found in the hindgut (Sawaya et al. 2008). França and Araújo (2006) suggested that it is a habitat and diet specialist; however, it was found in urban areas by São Pedro and Pires (2009). All individuals were observed during the wet season, between December and March. Among the females examined, two had ovarian follicles, the first (MRCM 427) having seven (LOF = 9.7 mm) in February and the second (MRCM 11), 37 (LOF = 9 mm) in March. The female reproductive cycle is seasonal (vitellogenesis between September and April and egg laying between November and April) and fecundity can vary from two to four eggs (Resende and Nascimento 2015). According to Sawaya et al. (2008), the species is more active on the surface during the wet season, mainly between October and February (mainly October). It reaches sexual maturity with at least 247 mm SVL in females and 187 mm in males (Resende and Nascimento 2015). When handled, it can press the sharp tip of the tail against the captor’s hand (N = 1). The available information indicates that it can perform erratic movements or produce cloacal discharge (Marques et al. 2015; Table 4).

***Dipsasmikanii* Schlegel, 1837**

Figure 6D

It is a medium-sized aglyphous species (mean SVL = 270 mm; range 122–465 mm; N = 36; França et al. 2008). Only two individuals were found (OLP), one of them moving during the day on turf in disturbed areas (MRCM 1288 near the SBES headquarters, and the other, MRCM 475, in a vegetable garden next to a residence). The available information indicates that it is terrestrial and primarily nocturnal (Sazima and Manzini 1995; França and Araújo 2006; Sawaya et al. 2008) and that it can be found in forest and disturbed areas (Sawaya et al. 2008). It feeds on gastropods, which, probably due to the low caloric value, are ingested in large numbers in a short period of time (Sazima and Manzini 1995; França and Araújo 2006; Barbo et al. 2011). One of the individuals (MRCM 1288) observed (in June, dry season) was a new-born. The species has a seasonal reproductive cycle (during the hottest and wettest months of the year) and clutch size varies from three to ten eggs (Pizzatto et al. 2008). There are reports of communal nests for this species (Albuquerque and Ferrarezzi 2004; Braz et al. 2008). When handled, it can compress the body while raising the head (MRCM 1288; see Fiorillo et al. 2019), triangulate the head, and perform erratic movements (MRCM 1288). It can also hide its head or produce cloacal discharge (Marques et al. 2015).

***Erythrolamprusaesculapii* (Linnaeus, 1758)**

Figure 6E

It is a medium-sized opisthoglyphous species (mean SVL = 598 mm; range 580–615 mm; N = 3; this study). Most individuals (AE = 1, OLP = 1) were observed close to forest areas (MRCM 361 and a specimen not collected in cerradão and MRCM 252 in a transition area between a pine forest and cerradão); the fourth individual (MRCM 424) was captured (OLP) in a disturbed area near the SBES headquarters. Two individuals were observed moving during the day (between 09:00 AM and 09:30 AM; MRCM 252 and MRCM 361). The available information indicates that the species is terrestrial, cryptozoic, and diurnal (Bernarde and Abe 2006; França and Araújo 2006; Martins et al. 2008). Two specimens examined had contents in their digestive tracts: one contained hair (MRCM 424; probably secondary ingestion) in the hindgut and the other (MRCM 361), snake scales. This species feeds mainly on other snakes (Martins and Oliveira 1998; Bernarde and Abe 2006; Marques et al. 2015, Fiorillo et al. 2020a). The individuals observed were captured in both the dry and wet seasons (during the months of January, May, July, and December). One female collected in January (MRCM 424) had five ovarian follicles (LOF = 7.6 mm). The available information indicates that its fecundity varies from three to eight eggs and it has a continuous reproductive cycle, with vitellogenesis throughout the entire year; however, ovarian follicles (including eggs) are larger during the wet season (October–April; Marques 1996; Marques and Sazima 2004). It reaches sexual maturity with at least 635 mm SVL in females and 430 mm in males (Marques 1996). When handled, it can flatten its body (N = 2; MRCM 361, 424), raise the curled tail as in *Micrurusfrontalis* (N = 3; MRCM 252, 361, 424), bite (N = 2; MRCM 252, 361), produce cloacal discharge (N = 1; MRCM 252) or erratic movements (N = 1; MRCM 361) (Table 4). Furthermore, besides being a supposed mimic of elapids (e.g., *Micrurusfrontalis*), this species can also hide its head (Martins et al. 2008; Marques et al. 2015) and compress its body while raising its head (Greene 1979). An envenomation by this species at the SBES (MRCM 361) was described by Menegucci et al. (2019).

***Erythrolamprusalmadensis* (Wagler, 1824)**

Figure 6F

It is a small aglyphous species (it can reach just longer than 500 mm SVL; Dixon 1991). The only individual observed (MRCM 1289) was captured by PT, in a campo sujo area. In addition to open habitats, it can also occur in forests and even disturbed areas (Strüssmann and Sazima 1993; Carvalho and Nogueira 1998; Bernarde and Abe 2006). It is semi-aquatic and diurnal (Bernarde and Abe 2006; França and Araújo 2006; Marques et al. 2015), and feeds primarily on amphibians (Bernarde and Abe 2010; França and Araújo 2006; Marques et al. 2015). The individual found was a new-born (SVL = 185 mm) and was captured in July (dry season). Its fecundity varies from four to ten eggs (Achaval and Olmos 1997). When handled, it can produce cloacal discharge (Marques et al. 2015; Table 4).

***Erythrolampruspoecilogyrus* (Wied-Neuwied, 1825)**

Figure 7A

It is a small aglyphous species (mean SVL = 359 mm; range 275–429 mm; N = 3; this study). Three individuals were observed in open areas (campo cerrado, cerrado sensu stricto, and one in a disturbed area at the SBES headquarters; MRCM 369, 1277, and 241, respectively). One (MRCM 241) was accidentally found moving during the day (04:00 PM) and another (MRCM 369) during TCS at night (08:05 PM). The other was captured though AE. The available information indicates that the species is terrestrial and both diurnal and nocturnal (this study; França and Araújo 2006; Martins et al. 2008; Marques et al. 2015). A study carried out on the north coast of Rio Grande do Sul revealed that the species shows a bimodal activity pattern throughout most of the year (early morning and late afternoon), except in the coldest months when it is active in the hottest periods of the day (Maciel et al. 2003). The available information indicates that it feeds mainly on amphibians (Pinto and Fernandes 2004; França and Araújo 2006; Marques et al. 2015). All individuals observed in this study were captured during the wet season, between October and April. It has a continuous reproductive cycle (Maciel et al. 2003) and fecundity varies from three to 15 eggs, with births in January and February (Achaval and Olmos 1997; Pinto and Fernandes 2004; Sawaya et al. 2008). In Argentina, males reach sexual maturity with at least 211 mm SVL and females with 250 mm (Prieto et al. 2012). When handled, it can produce cloacal discharge (N = 1; MRCM 241) and can also flatten or twist its body (Martins et al. 2008; Sawaya et al. 2008; Marques et al. 2015) (Table 4).

***Erythrolamprusreginae* (Linnaeus, 1758)**

Figure 7B

It is a medium-sized aglyphous species (mean SVL = 417 mm; range 190–597 mm; N = 19; França et al. 2008). All individuals (N = 4) were observed in the cerradão and captured by PT. As all individuals were captured in PTs, information on micro-habitat use or daily activity was not obtained. Among four specimens examined, only one (MRCM 368) had gastrointestinal contents, one anuran (*Physalaemus* sp.) in the stomach and lizard scales in the hindgut. The available information indicates that it is terrestrial and both diurnal and nocturnal (Martins and Oliveira 1998; Bernarde and Abe 2006; França and Araújo 2006) and feeds primarily on frogs, but occasionally lizards and invertebrates (Martins and Oliveira 1998; Bernarde and Abe 2006; França and Araújo 2006; Albarelli et al. 2010). The four individuals sampled were captured between September (late dry season) and March (wet season). A female collected in February (MRCM 434) had six ovarian follicles (LOF = 16.5 mm). The available information indicates that the species has a fecundity varying from three to six eggs (Duellman 1978). Martins and Oliveira (1998) suggested that births occur throughout the year in the Amazon. When handled, it can produce cloacal discharge (N = 2; MRCM 434, 1265), flatten the body (N = 2; MRCM 508, 1265), or perform erratic movements as previously described for the species (Marques et al. 2015) (Table 4).

***Oxyrhopusguibei* Hoge & Romano, 1978**

Figure 7C

It is a medium-sized opisthoglyphous species (mean SVL = 551 mm; range 415–835 mm; N = 4; this study). The individuals were found in campo cerrado (AE = 4, PT = 3, TCS = 1; MRCM 326, 431, 1388), campo sujo (PT = 4; MRCM 325, 466, 1000, 1183), cerrado sensu stricto (PT = 2; MRCM 344, 1276), in a wet field near a campo cerrado area (AE = 1; MRCM 1216) and in disturbed areas, close to an area of cerradão (OLP = 1; MRCM 217). Three individuals were moving on the ground at night (between 07:00 PM and 08:00 PM; MRCM 326, 1216, and other individual). The available information indicates that it is terrestrial and nocturnal (França and Araújo 2006; Sawaya et al. 2008). One individual regurgitated a rodent (MRCM 217) and two (an adult and a juvenile; MRCM 1000 and 1183, respectively) caught in PT regurgitated lizards of the species *Aspronemadorsivittatum*. Among the eight other specimens examined, four (MRCM 325, 431, 466, 1183) had ingested lizards. The available information indicates that it feeds on lizards and mammals (Sazima and Haddad 1992; França and Araújo 2006; Marques et al. 2015). All observed individuals were captured between August (dry season) and April (late wet season). One female collected in October (MRCM 326) had seven ovarian follicles (LOF = 27.7 mm). This species presents a continuous reproductive cycle, with recruitment mainly in the late wet season and early dry season; males reach sexual maturity with at least 388 mm SVL and females with 632 mm (Pizzatto and Marques 2002). When handled, it can perform erratic movements (N = 3; MRCM 1000), produce cloacal discharge (N = 1), and raise a curled tail as in *Micrurusfrontalis* (N = 1; MRCM 1000). The available information indicates that it can also hide its head or twist the body (Sawaya et al. 2008; Marques et al. 2015) (Table 4).

***Oxyrhopusrhombifer* Duméril, Bibron & Duméril, 1854**

Figure 7D

It is a medium-sized opisthoglyphous species (mean SVL = 447 mm; Cunha and Nascimento 1983; Gaiarsa et al. 2013), and can reach 958 mm SVL (Giraudo 2001). Only a juvenile was observed in this study, in the campo sujo (captured through PT). It is terrestrial and nocturnal (França and Araújo 2006; Sawaya et al. 2008). The available information indicates that it feeds mainly on lizards and mammals, but occasionally on snakes (França and Araújo 2006; Gaiarsa et al. 2013). The only individual sampled was captured in March (wet season) and was a new-born. According to the literature, its fecundity varies from four to 17 eggs (Pontes and Di-Bernardo 1988; Yanosky et al. 1996; Gallardo and Scrocchi 2006; Sawaya et al. 2008; Gaiarsa et al. 2013) and it reaches sexual maturity with at least 452 mm SVL in males (Gaiarsa et al. 2013) and 442 mm in females (Cunha and Nascimento 1983). When handled, it can flatten or twist its body, hide its head, perform erratic movements, or produce cloacal discharge (Marques et al. 2015) (Table 4).

***Phalotrislativittatus* Ferrarezzi, 1993**

Figure 7E

A medium-sized opisthoglyphous species (mean SVL = 758 mm; range 518–1,222 mm; N = 3; this study) found in campo cerrado (AE = 1, PT = 6; MRCM 317, 315, 406, 518, 535), cerrado sensu stricto (PT = 3; MRCM 322, 316, 1222), edge of cerradão (TCS = 1; MRCM 370), and in a disturbed area near to the SBES headquarters (OLP = 1; MRCM 200). One of the individuals was observed moving at night (MRCM 370). The available information indicates that it is fossorial and nocturnal (Sawaya et al. 2008; Marques et al. 2015). One specimen (MRCM 200) contained fragments of an amphisbaenian in the hindgut and in another, scales of the snake *T.koppesi* also in the hindgut (MRCM 406). It feeds on elongated vertebrates like amphisbaenians and snakes (this study; Braz et al. 2014; Marques et al. 2015). All individuals from this study were found during the wet season, between October and February. A female (MRCM 1222) collected in February had an ovarian follicle (7.4 mm). According to the literature, it can be found active in the wet season, from October to February, when females may be reproductive (Sawaya et al. 2008; Braz et al. 2014). It reaches sexual maturity with at least 507 mm SVL in females and 409 mm in males (Braz et al. 2014). When handled, it can press the sharp tip of the tail against the captor’s hand or perform erratic movements (Marques et al. 2015) (Table 4).

***Phalotrismertensi* (Hoge, 1955)**

Figure 7F

It is a large opisthoglyphous species (mean SVL = 802 mm; range 304–1262 mm; N = 50; Sawaya et al. 2008). Only a juvenile (MRCM 1296) was accidentally encountered (AE) in a cerradão area, while apparently thermoregulating in a sunspot during the day (01:00 PM). It can also use open habitats such as campo sujo and disturbed areas (Sawaya et al. 2008; Araujo et al. 2010). The available information indicates that it is fossorial and can present both diurnal and nocturnal activity (Sawaya et al. 2008). It is probably specialised in feeding on amphisbaenians and other elongated vertebrates with fossorial habits, like other elapomorphines (cf. Savitzky 1979). The only individual from this study was found in July (dry season). Its reproductive cycle is seasonal, with secondary vitellogenesis occurring between August and February; it can present eggs in oviducts in December, indicating that copulation occurs around November (Sawaya et al. 2008). Males reach sexual maturity with a minimum of 481 mm SVL and females with 513 mm, and its fecundity varies from three to seven eggs (Sawaya et al. 2008). When handled, it can open its mouth wide (N = 1, see Fiorillo et al. 2018) and perform erratic movements (N = 1). It can also press the sharp tip of the tail against the captor’s hand (Marques et al. 2015) (Table 4).

***Philodryasolfersii* (Lichtenstein, 1823)**

Figure 8A, B

It is a large, opisthoglyphous species (mean SVL = 699 mm; range 285–1120 mm; N = 129; Sawaya et al. 2008). Two individuals (including MRCM 1255) were accidentally encountered (AE) on the same day, both active during the day in the cerradão; one of them had ingested a rodent inside one of the pitfall traps and the other was moving around in the leaf litter. A third individual (juvenile; MRCM 1304) found on the edge of a cerradão area after the end of the study contained an anuran (*Scinaxfuscovarius*) in its stomach. It is semi-arboreal, diurnal (Bernarde and Abe 2006; Hartmann and Marques 2005; Martins et al. 2008), and feeds mainly on amphibians and mammals (Hartmann and Marques 2005; Marques et al. 2015). The first two individuals (the juvenile was found in August) from this study were observed in March (wet season) and one female (MRCM 1255) had 22 ovarian follicles (LOF = 11 mm). It presents a seasonal reproductive cycle, with secondary vitellogenesis occurring between January and May, and ovulation and oviposition occurring between September and January (Fowler et al. 1998; Mesquita et al. 2013b); its fecundity varies from four to 11 eggs (Achaval and Olmos 2008; Mesquita et al. 2013b). It reaches sexual maturity with approximately 490 mm SVL in males and 640 mm in females (Mesquita et al. 2013b). When handled, it can open its mouth wide, strike, form an S-coil with the anterior part of its body, or produce cloacal discharge (Martins et al. 2008; Marques et al. 2015) (Table 4).

***Philodryaspatagoniensis* (Girard, 1858)**

Figure 8C

A large, opisthoglyphous species (mean SVL = 696 mm; range 205–1120 mm; N = 140; Sawaya et al. 2008), reaching 1.315 mm SVL (López and Giraudo 2008). A single individual (MRCM 1287) was found in campo sujo, in a closed pitfall trap (PT). However, it can also be found in areas of denser vegetation such as cerrado sensu stricto (Araujo et al. 2010) and forest edges or in disturbed areas (Sawaya et al. 2008). It is terrestrial and diurnal (Di-Bernardo 1998; Hartmann and Marques 2005; Sawaya et al. 2008; Marques et al. 2015). *Philodryaspatagoniensis* feeds mainly on frogs and lizards, and may occasionally prey on other snakes, birds, and mammals (Hartmann and Marques 2005; López and Giraudo 2008). The only individual (an adult male) was captured in July (dry season). It has a seasonal reproductive cycle, with secondary vitellogenesis between August and December and ovulation between October and December (Fowler et al. 1998). Males reach sexual maturity with ca. 410 mm SVL and females with 550 mm and its fecundity varies from one to 26 eggs (Achaval and Olmos 1997; López and Giraudo 2008). When handled, it can bite, flatten or twist its body, produce cloacal discharge, or triangulate the head (Martins et al. 2008; Sawaya et al. 2008; Marques et al. 2015) (Table 4).

***Pseudoboanigra* (Duméril, Bibron & Duméril, 1854)**

Figure 8D, E

It is a large opisthoglyphous species (range 548–1261 mm; N = 110; Orofino et al. 2010), with a maximum SVL of 1261 mm (Gaiarsa et al. 2013). Two individuals were observed through TCS: one adult (MRCM 342) was observed on the edge of a gallery forest and another (a juvenile), under fallen eucalyptus trunks in campo sujo, both active at night. Under the trunks where the second individual was found, we also found two lizard species, *Aspronemadorsivittatum* and *Tropidurusitambere*. It can use forests and open areas and is able to persist in disturbed habitats (Gaiarsa et al. 2013). It is terrestrial and predominantly nocturnal (Guedes 2006; Gaiarsa et al. 2013). The individual captured at the edge of the gallery forest defecated lizard fragments. Although it feeds frequently on lizards, it can occasionally feed on other snakes, amphisbaenians, and even small mammals (Gaiarsa et al. 2013). The two individuals observed in this study were found during the wet season, in October (adult male) and November (juvenile female). Orofino et al. (2010) reported females with six to eight eggs in the oviducts in February and September. It reaches sexual maturity with ca. 560 mm SVL in females and 548 mm in males; its fecundity can vary from three to 24 eggs (Orofino et al. 2010). When handled, it can produce cloacal discharge, hide its head (MRCM 342), and can also perform erratic movements (Marques et al. 2015) (Table 4).

***Rhachidelusbrazili* Boulenger, 1908**

Figure 8F

It is a large, opisthoglyphous species (mean SVL = 786 mm; range 435–1200 mm; N = 6; França et al. 2008), reaching up to 1,372 mm SVL (Gaiarsa et al. 2013). One individual (IBSP89664) was observed (during TCS) in a transition between the campo sujo and a recently burned campo cerrado (very sparse vegetation), moving on the ground at night (07:40 PM). It is terrestrial and nocturnal, and appears to be a habitat generalist since it may use both open and forest habitats as well as disturbed areas (Sawaya et al. 2008; Gaiarsa et al. 2013). The captured individual defecated bird remains (bones and feathers). Most diet records for *R.brazili* are bird eggs, but it can occasionally feed on birds (this study; Gaiarsa et al. 2013). The captured individual was a sub-adult female and was found in November (wet season). It reaches sexual maturity with 984 mm SVL in females and 867 mm in males (Gaiarsa et al. 2013). When handled, the individual flattened its body, performed erratic movements, produced cloacal discharge, and triangulated its head (N = 1) (Table 4).

***Taeniophallusoccipitalis* (Jan, 1863)**

Figures 9A

A small aglyphous species (mean SVL = 269 mm; range 140–399 mm; N = 27; Sawaya et al. 2008) reaching 453 mm SVL (this study). Nine individuals were found in non-forest vegetation types: campo sujo (PT = 1, TCS = 1; MRCM 435, 510), campo cerrado (AE = 3, PT = 1; MRCM 532, 1281, 1387), and cerrado sensu stricto (PT = 3; MRCM 523, 1294). One of them (MRCM 435) was moving on a sandy soil at night (08:00 PM) and the other (MRCM 532) in campo cerrado at 10:20 AM. According to the literature, it is terrestrial and diurnal, and can be found within the leaf litter (Sawaya et al. 2008; Morato et al. 2011; Marques et al. 2015), and does not seem to use disturbed areas (Sawaya et al. 2008). Two specimens examined showed lizard fragments in their hindgut (MRCM 510, 532). The available information indicates that it feeds mainly on amphibians and lizards (Cechin 1999; Marques et al. 2009; 2015), but there is one record of ophiophagy (Balestrin 2008). A female (MRCM 510) collected in September (dry season) had 17 ovarian follicles (LOF = 8.7 mm). Two specimens collected in February and April (wet season) were new-borns. When handled, it can twist its body (N = 1; MRCM 435) and perform death feigning (N = 1; MRCM 1294; see Fiorillo et al. 2019). It can also flatten its body or produce cloacal discharge (Marques et al. 2015) (Table 4).

***Thamnodynasteshypoconia* (Cope, 1860)**

Figure 9B, C

It is a small, opisthoglyphous species (mean SVL = 387 mm; range 364–417 mm; N = 5; this study). One individual was found near the campo sujo and the others in open flooded areas, always on the ground or in humid undergrowth, usually near water bodies with calling anurans. All individuals were moving at night and captured through TCS (between 06:30 and 9:20 PM). The available information indicates that the species is semi-arboreal and nocturnal (Martins et al. 2008; Sawaya et al. 2008; Marques et al. 2015) and that its diet is specialised in amphibians (França and Araújo 2006; Marques et al. 2015). All individuals were captured between October and March (the wet season). Published data indicate that its reproductive cycle is non-seasonal, with secondary vitellogenesis occurring between January and September, ovulation between March and November, and embryos are present in females between May and November, after which births should occur; its fecundity varies from one to 12 hatchlings (Achaval and Olmos 1997; Sawaya et al. 2008). When handled, it can bite (N = 2; MRCM 221). It can also open its mouth, flatten or twist the body, strike, produce cloacal discharge, or triangulate its head (Sawaya et al. 2008; Marques et al. 2015) (Table 4).

***Xenodonmerremii* (Wagler, 1824)**

Figure 9D

It is a large aglyphous species (mean SVL = 868 mm; range 845–903 mm; N = 3; this study). One individual (MRCM 319) was AE, while moving over the leaf litter in the cerradão during the day (11:15 AM), the others (MRCM 423, 495) were OLP in disturbed areas near the SBES headquarters. The available information indicates that it is terrestrial and diurnal (Vitt 1983; Sawaya et al. 2008; Marques et al. 2015), with a diet restricted to amphibians (Jordão 1997; Marques et al. 2015). The individuals were observed in October, February (wet season), and July (dry season) in this study. A female (MRCM 423) collected in February had 26 ovarian follicles (LOF = 7.2 mm). Males reach sexual maturity with at least 486 mm SVL and females with 561 mm (Pizzatto et al. 2008). It has a continuous reproductive cycle and fecundity varies from four to 44 eggs (Vitt 1983; Vitt and Vangilder 1983; Pizzatto et al. 2008). When handled, it can flatten its body (N = 2; MRCM 319, 495), compress the body while raising the head (N = 1; MRCM 495), strike (N = 2; MRCM 319, 495), open its mouth (N = 1; MRCM 319, 495), or triangulate or hide the head (N = 1; MRCM 495) (Table 4). The available information indicates that, in addition to being a supposed mimic of *C.durissus*, it can also raise a curled tail (Marques et al. 2015).

***Xenodonnattereri* (Steindachner, 1867)**

Figure 9E

It is a small aglyphous species (mean SVL = 272 mm; range 135–442 mm; N = 73; Sawaya et al. 2008). One individual (MRCM 1749) was captured through AE in campo sujo and another (MRCM 517) was OLP on the ground, in an area of campo cerrado. It presents a wide distribution throughout the Cerrado and transitional zones with the Atlantic Forest (Nogueira et al. 2019). Sawaya et al. (2008) suggest that it is fossorial and terrestrial (its activity on the surface is virtually limited to the wet season) and diurnal. One individual (MRCM 517) defecated two lizard eggs. In addition to lizard eggs (this study; Sawaya et al. 2008), it can also prey on lizards (particularly from the family Gymnophthalmidae) and snakes (Sawaya et al. 2008). The female (MRCM 517) collected in October (wet season) had eight ovarian follicles (LOF = 3.8 mm). Its fecundity varies from two to ten eggs and Sawaya et al. (2008) suggest that it presents seasonal reproduction, with secondary vitellogenesis occurring between October and February. When handled, it can flatten its body, hide its head, or curl its tail (Marques et al. 2015) (Table 4).

#### Elapidae Boie, 1827

***Micrurusfrontalis* (Duméril, Bibron & Duméril, 1854)**

Figure 9F

It is a large proteroglyphous species (mean SVL highly variable according to the subspecies; range 500–1,425 mm; Roze 1996; Sawaya et al. 2008). The only individual (MRCM 489, a juvenile, SVL = 338 mm) found in this study was captured in a PT in campo cerrado. The available information indicates that it is terrestrial, fossorial, both nocturnal and diurnal, and apparently a habitat specialist of open habitats (Sazima and Abe 1991; França and Araújo 2006; Sawaya et al. 2008; Marques et al. 2015). It feeds on elongated vertebrates such as gymnophthalmid lizards, amphisbaenians, and other snakes, and there are reports of cannibalism (Roze 1996). The available information indicates that it is oviparous and presents a seasonal reproductive cycle, with a long period of vitellogenesis in the early wet season and mating from late wet season to the first half of the dry season (autumn) (Marques et al. 2006). When handled, the single individual performed erratic movements, raised a curled tail, showing it, and hid the head (Table 4). It can also flatten it body (Marques et al. 2015) and evert the hemipenes (Sazima and Abe 1991).

***Micruruslemniscatus* (Linnaeus, 1758)**

Figure 10A

It is a large proteroglyphous species (range 500–900 mm; Roze 1996). The only individual found (MRCM 1700, a juvenile, SVL = 317 mm) in this study was captured through AE in campo cerrado. The available information indicates that it is terrestrial, fossorial, both nocturnal and diurnal (Sazima and Abe 1991; Roze 1996), and can be observed in open vegetation types, disturbed areas, and forest edge (Sazima and Abe 1991). It feeds on elongated vertebrates, such as amphisbaenians, eels (*Gymnotus* or *Synbranchus*), and snakes (Sazima and Abe 1991; Roze 1996). The available information indicates that it is oviparous and its reproductive cycle is probably seasonal (Marques et al. 2013). When handled, the collected individual performed erratic movements, raised and curled the tail, or hid its head under body coils. It can also bite and flatten the body (Sazima and Abe 1991) (Table 4).

#### Viperidae Oppel, 1811

***Bothropsalternatus* Duméril, Bibron & Duméril, 1854**

Figure 10B

It is a large solenoglyphous species (mean SVL = 997 mm; range 930–1,070 mm; N = 4; this study), reaching 1,240 mm of CRC (Sawaya et al. 2008). Virtually all individuals were found in the most open vegetation types, such as campo sujo (AE = 7; MRCM 497) and campo cerrado (TCS = 1), and in disturbed areas near the SBES headquarters (OLP = 2). One individual was found in a recently burned campo cerrado area (with very sparse vegetation). Two adult females were found moving during the day (07:30 AM and 09:30 AM, respectively). One of them (MRCM 497, SVL = 1,000.5 mm, 660 g) regurgitated a freshly ingested rodent, *Clyomyslaticeps* (body length 190 mm, 295 g). Another adult female was found foraging (coiled in ambush posture) in a recently burned area of ​​campo cerrado at night (08:30 PM). The available information indicates that it is terrestrial, both diurnal and nocturnal (Martins et al. 2001; Sawaya et al. 2008; Marques et al. 2015; Fiorillo et al. 2020b), and is a mammal specialist (Martins et al. 2002). Most individuals were found between August (dry season) and March (wet season) and a female (MRCM 497) collected in August had 14 ovarian follicles (LOF = 13.7 mm). The reproductive cycle is seasonal, with births occurring probably between the middle and late wet season (Sawaya et al. 2008). Its fecundity varies from three to 25 young (Achaval and Olmos 1997). It reaches sexual maturity with at least 700 mm SVL in females and 440 mm in males (Nunes 2006). When handled, it can flatten the body (N = 2), strike (N = 3; MRCM 497), hide the head (N = 2; MRCM 497), or vibrate the tail (N = 2; MRCM 497) (Table 4).

***Bothropsitapetiningae* (Boulenger, 1907)**

Figure 10C

It is a small solenoglyphous species (mean SVL = 396 mm; range 185–570 mm; N = 89; Sawaya et al. 2008). It was found only in the most open areas of the SBES, in campo sujo (AE = 3) and in a recently burned area of ​​campo cerrado (TCS = 1; MRCM 366). One individual (MRCM 366) was found moving at night (07:35 PM) and another during the day (08:30 AM); both tried to escape by entering burrows in the ground. Two individuals were found coiled during the day (09:00 AM and 07:00 AM), one of them seemed to be foraging and the other was resting, sheltered under the lid of one of the PTs. The available information indicates it is nocturnal (occasionally diurnal) and terrestrial, specialised in open areas of the Cerrado and feeds on mammals, lizards, amphibians, birds, and centipedes (Martins et al. 2002; Sawaya et al. 2008; Leão et al. 2014; Marques et al. 2015; Fiorillo et al. 2020b). All individuals were found between November and March (the wet season) and one female (MRCM 366) collected in November had seven ovarian follicles (LOF = 3.9 mm). The available information indicates that it presents fecundity varying from three and 11 embryos with vitellogenic ovarian follicles occurring between January and June; births occur during the wet season (Sawaya et al. 2008; Leão et al. 2014). It reaches sexual maturity with at least 230 mm SVL in females and 340 mm in males (Leão et al. 2014). When handled, it can flatten the body (N = 1), strike (N = 1), or vibrate the tail (N = 2; MRCM 366) (Table 4). It can also open its mouth, hide its head, or produce cloacal discharge (Sawaya et al. 2008; Marques et al. 2015).

***Bothropsmoojeni* Hoge, 1966**

Figure 10D

It is a large solenoglyphous species (mean SVL = 880 mm; range 680–1,150 mm; N = 11; this study). It was generally found associated with flooded open areas and gallery forests (AE = 5, TCS = 9; MRCM 215, 338); it was also found in campo cerrado (TCS = 1; MRCM 293), cerrado sensu stricto (PT = 1) and cerradão (TCS = 1). Three individuals were found in disturbed areas (OLP = 2; MRCM 240). All individuals found in this study were active, moving, eating or in an ambush posture (tight coil and head raised at 45°), at night (between 07:30 and 11:00 PM), except for a single adult individual, OLP, moving during daytime (10:00 AM) (see also Fiorillo et al. 2020b). It is semi-arboreal and both diurnal and nocturnal (França and Araújo 2006; Sawaya et al. 2008). One individual was observed preying on a leptodactylid frog and another had rodent fragments in its faeces. The available information indicates that it presents ontogenetic changes in diet, feeding on ectothermic animals (amphibians, centipedes, lizards, and other snakes) when juvenile and on endothermic ones (mammals and birds) when adult (Brites 1992; Andrade et al. 1996; Martins et al. 2002; Nogueira et al. 2003; França and Araújo 2006; Ávila and Porfírio 2008). However, adults can occasionally prey on ectothermic vertebrates (e.g., Fiorillo et al. 2012). All individuals from this study were found between September (dry season) and March (wet season). A female (MRCM 338) collected in October (wet season) had 28 ovarian follicles (LOF = 5.2 mm) and a new-born was observed in September. Nogueira et al. (2003) suggest that their reproductive cycle is seasonal, and its fecundity varies from three to 32 young, with ovulation around July, and births probably concentrated between December and January. It reaches sexual maturity with at least 760 mm SVL in females and 590 mm in males (Nogueira et al. 2003). When handled, it can strike (N = 3; MRCM 240), hide the head (N = 1), bite (N = 1), and vibrate the tail (N = 4; MRCM 240) (Table 4). It can also open the mouth, flatten the body, or produce cloacal discharge (Sawaya et al. 2008; Marques et al. 2015).

***Bothropspauloensis* Amaral, 1925**

Figure 10E

It is a medium-sized solenoglyphous species (mean SVL = 611 mm; range 504–782 mm; N = 17; this study). Most individuals were found in open areas of campo sujo (AE = 6, TCS= 3; IBSP-90.262/sb0515) and campo cerrado (AE = 17, OLP = 1, PT = 1, TCS = 16; MRCM 256, 356, 425, 432, IBSP-90.262/sb0514), four in cerrado sensu stricto (AE = 2, TCS = 2; MRCM 1226), and only one (TCS) in the cerradão. Three individuals were found in disturbed areas (AE = 1, OLP = 2; MRCM 484) and four were observed OLP, without data on location or habitat (see also Fiorillo et al. 2020b). Approximately 50% of the individuals (including MRCM 484, 1024) were found active (moving or in ambush posture) between 05:30 and 10:30 PM and four were observed moving during the day between 08:00 and 12:00 PM. Three additional individuals (including MRCM 356) were apparently found resting under bucket lids of PTs between 07:00 and 09:30 AM. It is primarily nocturnal (Valdujo et al. 2002; Campbell and Lamar 2004; Jansen 2006), terrestrial (Marques et al. 2015), and can use burrows and other cavities (e.g., termite mounds) as sites to forage and shelter (Valdujo et al. 2002; Jansen 2006; Fiorillo et al. 2020b). One individual was found under fallen *Eucalyptus* trunks and bark, near a campo sujo area, where there were also lizards of the species *A.dorsivittatum* and *T.itambere*. Among the nine specimens examined, two adults had gut contents (an anuran of the genus *Leptodactylus* in the stomach and invertebrate remains in the hindgut, respectively). The available information indicates that it feeds on mammals, lizards, amphibians, centipedes, other snakes, and birds (Martins et al. 2002; Valdujo et al. 2002). Most of the individuals found active were captured between August (dry season) and March (wet season). The examined females had 11 (LOF = 6 mm) and 15 (LOF = 3.6 mm; MRCM 256) ovarian follicles in August and 18 (LOF = 4.5 mm; MRCM 425) in January. Three new-borns were found in January, February, and June. Valdujo et al. (2002) suggested that its reproductive cycle is seasonal, with vitellogenesis apparently starting ca. March, beginning embryo development from June to October, and the end of development in December; females give birth to 4–20 hatchlings per litter. It reaches sexual maturity with at least 475 mm SVL in females and 430 mm in males (Valdujo et al. 2002). When handled, it can flatten (N = 1) or twist its body (N = 1), strike (N = 16; MRCM 256, 356, 484, 507), hide its head (N = 1), and vibrate the tail (N = 12; MRCM 256, 356, 484, 507) (Table 4).

***Crotalusdurissus* (Linnaeus, 1758)**

Figure 10F

It is a large solenoglyphous species (mean SVL = 900 mm; range 825–988 mm; N= 12; this study). The individuals were observed in all vegetation types: campo sujo (AE = 3; MRCM 421, 1218), campo cerrado (AE = 2, TCS = 2) cerrado sensu stricto (AE = 3, OLP = 2), cerradão (AE = 10, OLP = 1, TCS = 1), in a transitional area between campo sujo and campo cerrado (AE = 1, TCS = 2) in a wet field (TCS = 1), on the edge of the gallery forest (AE = 1), and in disturbed areas (near farms outside the SBES, in disturbed areas at the SBES headquarters, and in pine forests; AE = 3, OLP = 4, TCS = 1; MRCM 289, 354, 392, 421, 485, 1273) (see also Fiorillo et al. 2020b). Five individuals OLP have no information on habitat. Of the individuals observed, eight were active at night (in ambush position or moving between 07:00 and 09:30 PM) and eight were moving during the day (between 09:00 AM and 03:00 PM). The available information indicates that it presents terrestrial habits and higher activity in the first hours of the night (Tozetti and Martins 2008; Tozetti et al. 2009; Marques et al. 2015). Although it is found predominantly in open and relatively dry areas (e.g., Sawaya et al. 2008), it has a great ecological plasticity and can be found even inside or at the edge of forests (this study; Reinert 1992; Bastos et al. 2005; Sawaya et al. 2008; Hartmann et al. 2009). In addition, it can use disturbed habitats and urbanised areas (Almeida-Santos and Orsi 2002; Sawaya et al. 2008; São Pedro and Pires 2009). Two individuals defecated hair and bones and a new-born (MRCM 485) regurgitated a rodent. Among seven specimens examined, three (MRCM 392, 485, 1107) contained hair in their hindgut. Its diet is specialised in mammals, but it can occasionally consume lizards (Almeida-Santos and Germano 1996; Salomão et al. 2005; Sant’anna and Abe 2007). Most adult individuals were found between November and March (wet season) and new-borns in April and May (dry season), and a female (MRCM 505) collected in August (dry season) had eight ovarian follicles (LOF = 4.3 mm). According to the literature, its activity is concentrated at the end of the wet season, when copulation and combat between males occur (Almeida-Santos and Orsi 2002). It is viviparous with prolonged vitellogenesis beginning in March, pregnancy between October and January, and juvenile recruitment between January and March (Almeida-Santos and Salomão 1997; Almeida-Santos and Orsi 2002). Its fecundity varies from 21 to 31 hatchlings (Vitt 1992). It reaches sexual maturity with at least 830 mm SVL in females and 820 mm in males (Barros et al. 2012). When handled, it can strike (N = 5; MRCM 505, 1218, 1273), vibrate the tail (N = 6; MRCM 505, 1273), or perform cloacal discharge (N = 2) (Table 4). It is also known to flatten the body (Sawaya et al. 2008).

## Discussion

This study has added 14 species to the previous list of snakes found at SBES (Araújo et al. 2010): *A.dimidiata*, *B.alternatus*, *B.itapetiningae*, *C.brazili*, *C.quadricarinatus*, *E.aesculapii*, *E.crassus*, *L.ternetzii*, *M.lemniscatus*, *P.lativittatus*, *P.olfersi*, *P.nigra*, *R.brazili*, and *X.merremi*. On the other hand, we failed to find two species listed by Araújo et al. (2010), *Philodryasagassizii* captured in the SBES during their study, and *Philodryaslivida*, which is known only from a historical record (Instituto Butantan collection number IBSP 40953) with unclear locality data that was attributed to the Municipality of Águas de Santa Bárbara by the authors. However, there are three specimens of *P.livida* collected in the 1970s in the records of the Instituto Butantan collection, collected from the Municipality of Lençois Paulista, the centre of which is located only 44 km from the SBES. Thus, this species may have occurred at SBES in the past (see discussion on conservation below). As for *P.agassizii*, it must have gone unnoticed in our study, despite the large sampling effort. Thus, considering this study and that of Araújo et al. (2010), 35 species of snakes occur in the SBES.

Approximately half of the species of snakes found at the SBES used non-forest vegetation types almost exclusively (campo sujo, campo cerrado, and/or cerrado sensu stricto). Other studies already showed a trend of decrease in snake species richness with increasing vegetation complexity in the Cerrado (Sawaya et al. 2008; Araujo et al. 2010; Araujo and Almeida-Santos 2011; Serrano-Filho 2012). The structural differences found between Cerrado vegetation types determine microclimatic differences at the local scale (e.g., Gianotti et al. 2013), with contrasting ranges of temperature and air humidity when the most open vegetation types (grasslands, the campos) are compared to the most closed ones (cerrado woodland, the cerradão). These different microclimates reflect differences in the solar and wind incidences between these habitats (e.g., Vitt et al. 2007; Gianotti et al. 2013) and may play a crucial role in determining snake diversity in the cerrado gradient of vegetation types, especially regarding heliophilic species.

The low proportion of species that use the arboreal strata (21%) in contrast to studies carried out in forests (e.g., Martins and Oliveira 1998; Bernarde and Abe 2006) is expected for a snake community from the Cerrado. Arboreal snakes seem to be less able to colonise non-forest formations (such as Cerrado and the southern Campos of Brazil; Cavalheri et al. 2015) due to the low availability of adequate substrates both currently and in the past (França et al. 2008; Cavalheri et al. 2015). Thus, more open vegetation types, such as those in the study area, could be working as an environmental filter (sensu Keddy 1992; Webb et al. 2002) for snake species of arboreal lineages (Cavalheri et al. 2015; Piatti et al. 2019).

The patterns of resources use described here for the snake community of SBES reflect mostly the composition of the community regarding snake lineages (Duellman 1978; Cadle and Greene 1993; Martins and Oliveira 1998; Bellini et al. 2015). In the SBES community, the closely related species were also more similar in terms of resource use as observed in other studies focusing on snake communities in the Neotropics (e.g., Duellman 1978; Martins and Oliveira 1998; Fiorillo et al. 2020a). For example, the species of the tribe Xenodontini (particularly the genera *Erythrolamprus* and *Xenodon*) are similar in terms of resource use, the same being the case for other lineages such as the tribe Pseudoboini and the families Boidae and Viperidae (see Table 1).

The most consumed prey items in the community (number of species which consumed a given prey type), based on the present study and the literature, were lizards followed by anurans and mammals. During our sampling, > 7,800 vertebrates were captured in pitfall traps, of which 64% were anurans, 16% were lizards, and 16% were small mammals. However, because of the highly seasonal rainfall in the Cerrado, frog activity is highly seasonal, with capture rates in pitfall traps increasing by orders of magnitude during the rainy season compared to the dry season (Brasileiro et al. 2005; Prado et al. 2005). Indeed, during the dry seasons (April to September) of the two years of sampling of this study, the proportion of anurans captured in PTs was 24% among vertebrates, while the capture rates of lizards and mammals were 37% each. The increased capture rates in the rainy season may reflect mostly the seasonal migrations of anurans to water bodies for breeding. On the other hand, the availability of lizards and small mammals tend to vary much less than that of amphibians. The decrease in anuran activity during the dry season may seasonally decrease the consumption rates of this item by snakes, and consequently increase that of lizards and mammals. In addition to the availability of different prey, the evolutionary history of each of the groups that make up the community also has a strong influence in relation to the prey items consumed (see above). For instance, within dipsadids, all species of the tribe Elapomorphini in the community feed on elongate vertebrates (mainly snakes and amphisbaenians); three of the four species of the tribe Pseudoboini feed primarily on mammals and lizards; most species of the tribe Xenodontini feed primarily on anurans; and both species of the tribe Philodryadini are diet generalists (Hartmann and Marques 2005; Sawaya et al. 2008).

Most reproductive females (ovarian follicles > 5 mm) were found from late dry season to late wet season. This peak of activity during the wet season in seasonal environments has also been recorded for other Neotropical snake communities (Strüssmann and Sazima 1993; Di-Bernardo 1998; Marques 1998; Sawaya et al. 2008; Pontes et al. 2009; Fiorillo et al. 2020a). Most juveniles (61.5%) were found from the late dry season to the onset of the wet season, which is also a recurring pattern (Marques 1998; Sawaya et al. 2008; Hartmann et al. 2009). In the Atlantic Forest, Hartmann et al. (2009) found more active snakes during the rainy/warmer season (October to March) than in the dry/colder season (April to September), but they did not find a significant difference between the number of snakes (adults or juveniles) captured. On the other hand, Sawaya (2004) detected significant correlations between the numbers of snakes captured and minimum and maximum temperatures in the Cerrado. That author also suggested that the minimum temperature probably would have a stronger effect on snakes than maximum temperature, since that variable limits snake activity (Lillywhite 1987). Despite the large number of studies that indicate seasonality in the reproductive cycles of tropical snakes, almost none attempted to demonstrate quantitatively the physiological bases that mediate this synchronisation. This is largely due to problems of discrimination between the effects of climate variables, their interactions, and their effects on the abundance of prey (Oliveira and Martins 2001; Mathies 2011).

Most of the defensive tactics observed in this study were supposedly directed at visually oriented predators (80% of tactics), such as birds (especially birds of prey), probably the most important predators of Neotropical snakes, and mammals (Martins 1996; Martins and Oliveira 1998; Martins et al. 2008). Indeed, most species also have cryptic colour patterns (ca. 80% of species). Among the species that show aposematic colour patterns (*A.dimidiata*, *E.aesculapii*, *M.frontalis*, *M.lemniscatus*, *O.guibei*, and *O.rhombifer*), four have fossorial and/or cryptozoic habits (*A.dimidiata*, *E.aesculapii*, *M.frontalis*, and *M.lemniscatus*) and the others, despite being terrestrial, are predominantly nocturnal (*O.guibei* and *O.rhombifer*). The great diversity of visual defensive behaviours associated with cryptic lifestyles and colour patterns is a common pattern in Neotropical snake communities and occurs in both predominantly open environments (e.g., this study; Sawaya et al. 2008) and forests (Martins and Oliveira 1998; Martins et al. 2008; Fiorillo et al. 2020a). Another defensive behaviour frequently used by SBES snakes was cloacal discharge, which in turn seems to be frequent in most species of snakes in different types of environment (see Martins and Oliveira 1998; Marques et al. 2005; Sawaya et al. 2008; Marques et al. 2015, 2017, 2019). The sharing of potential predators may have led to the widespread convergence of defensive tactics in snakes (Martins 1996; Martins et al. 2008), although some of these tactics are more phylogenetically conserved (e.g., the behaviour of inflating the gular region in colubrids; Martins et al. 2008).

The SBES snake fauna includes habitat specialists, sensitive to environmental disturbance, which are under different degrees of threat in the state of São Paulo. Four of these species are listed as threatened (Vulnerable, VU, or Endangered, EN) in the state of São Paulo red list (Governo do Estado de São Paulo 2018), *B.itapetiningae* (EN) *O.rhombifer* (VU), *P.agassizii* (EN) and *X.nattereri* (EN). Two species are listed as Near Threatened (NT) in the Brazilian red list (ICMBio 2018), *Phalotrislativittatus* and *B.itapetiningae*, whereas the former is listed as NT also in the IUCN red list (IUCN 2020). Additionally, *P.livida*, a species listed as VU in the three red lists cited above, is suspected to have occurred in the region of the SBES in the past (Araújo et al. 2010; see above). All these species are specialised in the open vegetation types of the Cerrado (especially the campos), are not able to persist in disturbed habitats (Sawaya et al. 2008) and seems not to be abundant in the study area. Unfortunately, one of the main conservation concerns regarding the rare fragments of cerrado in the southern portion of this biome is woody encroachment, which occurs in open vegetation types of many areas (Durigan et al. 2020). Thus, the future of the populations of the species above at SBES depends on a well-planned fire management program aiming at maintaining the open vegetation types in the long run (see recommendations in Durigan et al. 2020). It is worth noting that the local extinction of predators like snakes can lead to changes in the trophic structure of communities, consequently affecting several ecosystem functionalities (Estes et al. 2011).

## Conclusions

The Cerrado is the Neotropical Ecoregion with the greatest snake species richness (N = 222), with an average of 30 to 60 species every 12,000 km^2^ (1° × 1°) (Guedes et al. 2018). The SBES concentrates an important portion of this diversity in a small area (31.5 km^2^). The present study adds 14 species to the previous local snake list (Araujo et al. 2010). This reflects the importance of a complement of sampling methods as used in the present study, as well as the large sampling effort (23,040 buckets-days and 1,248 person-hours of time-constrained search in addition to accidental encounters and observations made by local people during four years of fieldwork at the reserve (see a similar example in Sawaya et al. 2008). Our study also provides a large amount of primary information about the species, which can assist in both understanding the structure of snake communities and contribute to the management and conservation practices in Cerrado areas. It is important to highlight the fact that the SBES contains one of the last remaining fragments of Cerrado in the state of São Paulo (Kronka et al. 2005), which in turn harbour many typical Cerrado snakes, including threatened and near threatened species (see above). Therefore, this area is of great value for the conservation of the Cerrado snake fauna.

## Appendix 1

Specimens examined; field number acronyms mean Marcio Roberto Costa Martins (the senior author’s full name). All these specimens will be deposited in the herpetological collections of the Museu de Zoologia da Universidade de São Paulo (MZUSP) and the Instituto Butantan

*Apostolepisdimidiata* (10) – Brazil: ÁGUAS DE SANTA BÁRBARA: Estação Ecológica de Santa Bárbara, MRCM 1256, 1702, 1802, 387, 463, 483, 512, 524, 541, 545.

*Atractuspantostictus* (4) – Brazil: ÁGUAS DE SANTA BÁRBARA: Estação Ecológica de Santa Bárbara, MRCM 11, 100, 389, 427.

*Boaconstrictor* (1) – Brazil: ÁGUAS DE SANTA BÁRBARA: Estação Ecológica de Santa Bárbara, MRCM 386.

*Bothropsitapetiningae* (1) – Brazil: ÁGUAS DE SANTA BÁRBARA: Estação Ecológica de Santa Bárbara, MRCM 366.

*Bothropsalternatus* (1) – Brazil: ÁGUAS DE SANTA BÁRBARA: Estação Ecológica de Santa Bárbara, MRCM 497.

*Bothropsmoojeni* (4) – Brazil: ÁGUAS DE SANTA BÁRBARA: Estação Ecológica de Santa Bárbara, MRCM 215, 240, 293, 338.

*Bothropspauloensis* (11) – Brazil: ÁGUAS DE SANTA BÁRBARA: Estação Ecológica de Santa Bárbara, IBSP-90.262/sb0514–515, MRCM 248, 256, 356, 425, 432, 484, 507, 1024, 1226.

*Chironiusbrazili* (1) – Brazil: ÁGUAS DE SANTA BÁRBARA: Estação Ecológica de Santa Bárbara, MRCM 482.

*Chironiusquadricarinatus* (1) – Brazil: ÁGUAS DE SANTA BÁRBARA: Estação Ecológica de Santa Bárbara, MRCM 1275.

*Crotalusdurissus* (10) – Brazil: ÁGUAS DE SANTA BÁRBARA: Estação Ecológica de Santa Bárbara, MRCM 289, 354, 392, 421, 485, 505, 1107, 1218, 1273, 1806.

*Dipsasmikanii* (2) – Brazil: ÁGUAS DE SANTA BÁRBARA: Estação Ecológica de Santa Bárbara, MRCM 475, 1288.

*Epicratescrassus* (2) – Brazil: ÁGUAS DE SANTA BÁRBARA: Estação Ecológica de Santa Bárbara, MRCM 359, 395.

*Erythrolamprusaesculapii* (1) – Brazil: ÁGUAS DE SANTA BÁRBARA: Estação Ecológica de Santa Bárbara, MRCM 252, 361, 424.

*Erythrolamprusalmadensis* (1) – Brazil: ÁGUAS DE SANTA BÁRBARA: Estação Ecológica de Santa Bárbara, MRCM 1289.

*Erythrolampruspoecilogyrus* (3) – Brazil: ÁGUAS DE SANTA BÁRBARA: Estação Ecológica de Santa Bárbara, MRCM 241, 369, 1277.

*Erythrolamprusreginae* (4) – Brazil: ÁGUAS DE SANTA BÁRBARA: Estação Ecológica de Santa Bárbara, MRCM 368, 434, 508, 1265.

*Liotyphlopsternezii* (2) – Brazil: ÁGUAS DE SANTA BÁRBARA: Estação Ecológica de Santa Bárbara, MRCM 433, 458.

*Micrurusfrontalis* (1) – Brazil: ÁGUAS DE SANTA BÁRBARA: Estação Ecológica de Santa Bárbara, MRCM 489.

*Micruruslemniscatus* (1) – Brazil: ÁGUAS DE SANTA BÁRBARA: Estação Ecológica de Santa Bárbara, MRCM 1700.

*Oxyrhopusguibei* (12) – Brazil: ÁGUAS DE SANTA BÁRBARA: Estação Ecológica de Santa Bárbara, MRCM 217, 249, 325, 326, 344, 431, 466, 1000, 1183, 1216, 1276, 1388.

*Oxyrhopusrhombifer* (1) – Brazil: ÁGUAS DE SANTA BÁRBARA: Estação Ecológica de Santa Bárbara, not labeled.

*Phalotrislativittatus* (10) – Brazil: ÁGUAS DE SANTA BÁRBARA: Estação Ecológica de Santa Bárbara, MRCM 200, 315, 316, 317, 322, 370, 406, 518, 535, 1222.

*Phalotrismertensi* (1) – Brazil: ÁGUAS DE SANTA BÁRBARA: Estação Ecológica de Santa Bárbara, MRCM 1296.

*Philodryasolfersii* (2) – Brazil: ÁGUAS DE SANTA BÁRBARA: Estação Ecológica de Santa Bárbara, MRCM 1255, 1304.

*Philodryaspatagoniensis* (1) – Brazil: ÁGUAS DE SANTA BÁRBARA: Estação Ecológica de Santa Bárbara, MRCM 1287.

*Pseudoboanigra* (1) – Brazil: ÁGUAS DE SANTA BÁRBARA: Estação Ecológica de Santa Bárbara, MRCM 342.

*Rachidelusbrazili* (1) – Brazil: ÁGUAS DE SANTA BÁRBARA: Estação Ecológica de Santa Bárbara, IBSP 89664.

*Taeniophallusoccipitalis* (7) – Brazil: ÁGUAS DE SANTA BÁRBARA: Estação Ecológica de Santa Bárbara, MRCM 435, 510, 523, 532, 1281, 1294, 1387.

*Tantillamelanocephala* (7) – Brazil: ÁGUAS DE SANTA BÁRBARA: Estação Ecológica de Santa Bárbara, MRCM 274, 291, 345, 461, 514, 544, 1298.

*Thamnodynasteshypoconia* (4) – Brazil: ÁGUAS DE SANTA BÁRBARA: Estação Ecológica de Santa Bárbara, MRCM 221, 222, 339, 534.

*Trilepidakoppesi* (24) – Brazil: ÁGUAS DE SANTA BÁRBARA: Estação Ecológica de Santa Bárbara, MRCM 290, 292, 294, 307–314, 318, 321, 323, 327, 328, 340, 355, 381–383, 457, 546, 1375.

*Xenodonmerremii* (3) – Brazil: ÁGUAS DE SANTA BÁRBARA: Estação Ecológica de Santa Bárbara, MRCM 319, 423, 495.

*Xenodonnattereri* (2) – Brazil: ÁGUAS DE SANTA BÁRBARA: Estação Ecológica de Santa Bárbara, MRCM 517, 1749.

## Supplementary materials

Supplementary material 1 Sampling efficiency evaluation Data type Analyses Explanation note **Figure S1.** Rarefaction curve representing the snake species richness observed (A) and the richness estimated by the Jackknife-1 estimator (B) for all methods together, and for each method used, accidental encounters (C), observations by local people (D), pitfall traps (E), and time constrained search (F) at the Santa Bárbara Ecological Station, state of São Paulo, Brazil. **Table S1.** Snake species richness (SR), number of individuals (Ind.), number of exclusive species, estimated richness of first order Jackknife, Shannon diversity index and dominance of each method used. **Table S2.** Number of snake individuals obtained by sampling method at the Santa Bárbara Ecological Station, SP, between August 2016 and July 2018. https://binary.pensoft.net/file/579235This dataset is made available under the Open Database License (http://opendatacommons.org/licenses/odbl/1.0/). The Open Database License (ODbL) is a license agreement intended to allow users to freely share, modify, and use this Dataset while maintaining this same freedom for others, provided that the original source and author(s) are credited.Bruno F. Fiorillo, Jorge Henry Maciel, Marcio Martins
