# Supplementary material for: Composition and natural history of a snake community from the southern Cerrado, southeastern Brazil
Source: Zookeys. 2021 Aug 19;1056:95–147. doi: 10.3897/zookeys.1056.63733 (PMC8390458; doi:10.3897/zookeys.1056.63733)
Supplement: Supplementary material 1 — Sampling efficiency evaluation [file zookeys-1056-095-s001.zip › 63733_0R-1-A_Supplementary material 1.docx]

**Supplementary material 1**

The method responsible for capturing the largest number of species was accidental encounters (AE; 21 species), while that responsible for capturing the largest number of individuals was pitfall trap (PT; 156 individuals) (Figure S1, Table S1). The latter method also resulted in the highest number of species captured exclusively with the method (Table S1). The number of species estimated to be captured by each method through rarefaction (considering the minimum number of individuals captured by a given method, 40 observations made by local people) was 13.92 ± 2.18 for AE, followed by OLP (N = 13), time-constrained search (TCS; 10.97 ± 2.18 species) and PT (9.10 ± 2.01). Moreover, AE remained the most efficient method according to the richness estimated by the Jackknife-1 estimator, followed by TCS and PT (both with 22.97 species), and OLP (Table S1). The most captured species (dominance) for three of the methods (AE, OLP, and TCS) was *Bothrops pauloensis* while *Trilepida koppesi* was the most captured through PT (Table S1).

The efficiency of each method varied according to the species (Table S2), with most dipsadids, *Liotyphlops ternetzii*, *Micrurus frontalis* (only one individual found during the entire sampling), *Tantilla melanocephala* and *Trilepida koppesi* being found through PT and boids and viperids through AE and TCS. Some species were captured exclusively by one method including: *Erythrolamprus almadensis*, *Erythrolamprus reginae*, *Micrurus frontalis*, *Oxyrhopus rhombifer*, and *Philodryas patagoniensis* (PT); *Chironius quadricarinatus*, *M. lemniscatus*, *Phalotris mertensi*, and *Philodryas olfersii* (AE), *Pseudoboa nigra*, *Rachidelus brazili* and *Thamnodynastes hypoconia* (TCS); *Chironius brazili* and *Xenodon nattereri* (OLP).

None of rarefaction curves reached an asymptote (including that of SBES as a whole, Figure S1), indicating that there is still species to be found through each of the methods used.

**Figures**

**Figure S1.** Rarefaction curve representing the snake species richness observed (A) and the richness estimated by the Jackknife-1 estimator (B) for all methods together, and for each method used, accidental encounters (C), observations by local people (D), pitfall traps (E), and time constrained search (F) at the Santa Bárbara Ecological Station, state of São Paulo, Brazil. Areas around the curve delimited by a dashed line represent 95% confidence intervals.


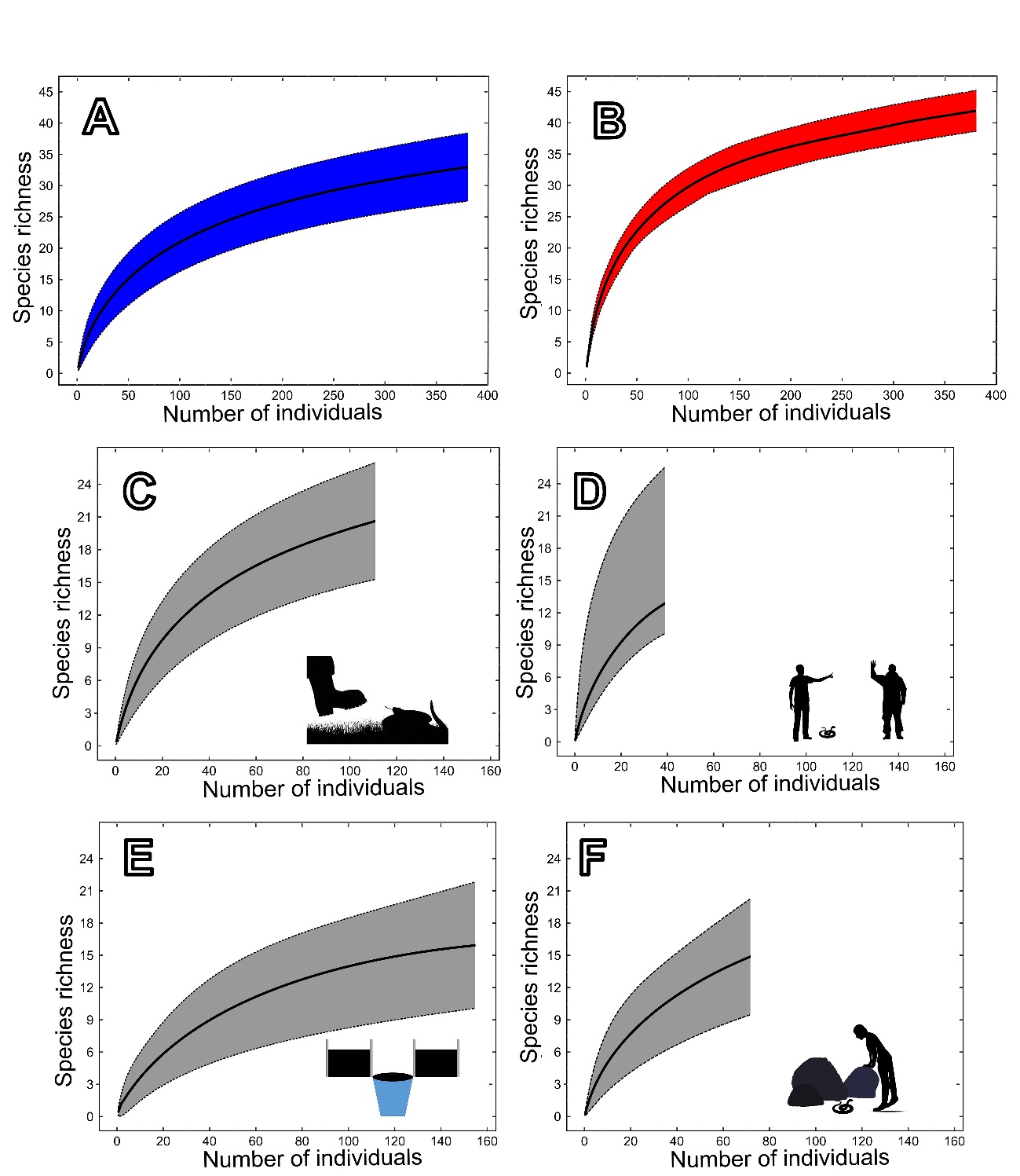


**Table S1.** Snake species richness (SR), number of individuals (Ind.), number of exclusive species, estimated richness of first order Jackknife, Shannon diversity index and dominance of each method used. AE = accidental encounters (specimens obtained by researchers in activities other than pitffall traps and visual search); OLP = observations by local people; PT = pitfall traps; TCS = time constrained search.

| **Method** | **SR** | **Ind.** | **ES** | **Jackknife I** | **Shannon (H’)** | **Dominance (%)** |
| --- | --- | --- | --- | --- | --- | --- |
| AE | 21 | 119 | 4 | 27.98 ± 2.61 | 2.49 | 21.85 |
| OLP | 13 | 40 | 3 | 17.98 ± 2.21 | 2.16 | 17.50 |
| PT | 16 | 156 | 5 | 22.97 ± 2.60 | 1.41 | 67.31 |
| TCS | 15 | 73 | 3 | 22.97 ± 3.12 | 2.07 | 30.14 |

Table S2. Number of snake individuals obtained by sampling method at the Santa Bárbara Ecological Station, SP, between August 2016 and July 2018: AE = accidental encounters (specimens obtained by researchers in activities other than pitfall traps and visual search); OLP = observations by local people; PT = pitfall traps; TCS = time constrained search.

| **TAXON** | **METHOD** | | | | **TOTAL** |
| --- | --- | --- | --- | --- | --- |
|  | **AE** | **OLP** | **PT** | **TCS** |  |
| **ANOMALEPIDIDAE** |  |  |  |  |  |
| *Liotyphlops ternetzii* |  |  | 3 |  | **3** |
| **BOIDAE** |  |  |  |  |  |
| *Boa constrictor* | 3 | 6 |  |  | **9** |
| *Epicrates crassus* | 3 |  |  | 2 | **5** |
| **COLUBRIDAE** |  |  |  |  |  |
| *Chironius brazili* |  | 1 |  |  | **1** |
| *Chironius quadricarinatus* | 1 |  |  |  | **1** |
| *Tantilla melanocephala* | 5 |  | 6 |  | **11** |
| **DIPSADIDAE** |  |  |  |  |  |
| *Apostolepis dimidiata* | 8 | 1 | 5 |  | **14** |
| *Atractus pantostictus* |  |  | 3 | 1 | **4** |
| *Dipsas mikanii* |  | 2 |  |  | **2** |
| *Erythrolamprus almadensis* |  |  | 1 |  | **1** |
| *Erythrolamprus aesculapii* | 2 | 2 |  |  | **4** |
| *Erythrolamprus poecilogyrus* | 2 |  | 1 | 1 | **4** |
| *Erythrolamprus reginae* |  |  | 4 |  | **4** |
| *Oxyrhopus guibei* | 5 | 1 | 9 | 1 | **16** |
| *Oxyrhopus rhombifer* |  |  | 1 |  | **1** |
| *Phalotris lativittatus* | 1 | 1 | 9 | 1 | **12** |
| *Phalotris mertensi* | 1 |  |  |  | **1** |
| *Philodryas olfersii* | 2 |  |  |  | **2** |
| *Philodryas patagoniensis* |  |  | 1 |  | **1** |
| *Pseudoboa nigra* |  |  |  | 2 | **2** |
| *Rhachidelus brazili* |  |  |  | 1 | **1** |
| *Taeniophallus occipitalis* | 3 |  | 5 | 1 | **9** |
| *Thamnodynastes hypoconia* |  |  |  | 6 | **6** |
| *Xenodon merremi* | 1 | 2 |  |  | **3** |
| *Xenodon nattereri* | 1 | 1 |  |  | **2** |
| **ELAPIDAE** |  |  |  |  |  |
| *Micrurus frontalis* |  |  | 1 |  | **1** |
| *Micrurus lemniscatus* | 1 |  |  |  | **1** |
| **LEPTOTYPHLOPIDAE** |  |  |  |  |  |
| *Trilepida koppesi* | 16 |  | 105 | 15 | **136** |
| **VIPERIDAE** |  |  |  |  |  |
| *Bothrops alternatus* | 7 | 2 |  | 1 | **10** |
| *Bothrops itapetiningae* | 3 |  |  | 1 | **4** |
| *Bothrops moojeni* | 5 | 2 | 1 | 11 | **19** |
| *Bothrops pauloensis* | 26 | 7 | 1 | 22 | **56** |
| *Crotalus durissus* | 23 | 12 |  | 7 | **42** |
| **TOTAL** | **119** | **40** | **156** | **73** | **388** |
